# A Comparison of Chemotherapy Used with and without Apatinib for Patients with Ovarian Carcinoma Who Progressed after Standard Regimens: A Systematic Review and Meta-Analysis

**DOI:** 10.1155/2021/2292907

**Published:** 2021-11-03

**Authors:** Chao Hou, Zhuang-Zhuang Jiang, Bo Pan, Xiao-Chun Zhang, Yan-Qing Liu

**Affiliations:** ^1^College of Medicine, Yangzhou University, Yangzhou 225001, Jiangsu, China; ^2^The Key Laboratory of Syndrome Differentiation and Treatment of Gastric Cancer of the State Administration of Traditional Chinese Medicine, Yangzhou 225001, China; ^3^College of Medicine, Yangzhou Hospital of Traditional Chinese Medicine Affiliated to Nanjing University of Chinese Medicine, Yangzhou 225009, Jiangsu, China

## Abstract

**Objective:**

This meta-analysis was conducted to compare the therapeutic efficacy and clinical safety of the combination therapy of apatinib plus chemotherapy with that of chemotherapy alone in patients with refractory or recurrent ovarian carcinoma (OC).

**Methods:**

Relevant randomized controlled trials (RCT) or case-control studies (CCS) were identified by searching Chinese and English databases up to October 31, 2020. The risk of methodological bias tool and Newcastle–Ottawa scale (NOS) were used to assess trial quality. Pooled odds ratios (OR) and 95% confidence intervals (CI) were calculated to evaluate the therapeutic effects and adverse drug reactions. Subgroup analyses of study type, study sample size, dosage of apatinib, and chemotherapy regimen between treatment group and control group were performed. Publication bias was assessed by funnel plot symmetry, Begg–Mazumdar test, and Egger test. The robustness of our results was presented by removing the trial one by one.

**Results:**

Fifteen eligible studies covering 1,020 patients were included in this review and meta-analysis. Among these studies, 8 were RCTs, and 7 were CCSs. Compared with chemotherapy alone, apatinib plus chemotherapy significantly increased objective response rate (OR = 3.55; 95% CI 2.31 to 5.47), disease control rate (OR = 3.04; 95% CI 2.12 to 4.36), and overall survival (OR = 5.03; 95% CI 3.16 to 6.90).

**Conclusions:**

The combination treatment of apatinib plus chemotherapy provides better clinical benefits for OC patients when compared to chemotherapy alone and should be recommended for suitable patients with OC after the failure of standard regimens. However, further investigation into future large-scale prospective randomized research is still needed.

## 1. Introduction

Ovarian carcinoma (OC) is one of the common malignant carcinomas in women worldwide. In 2018 alone, over 295,000 women developed OC, of whom over 184,000 died globally [1]. Although surgery and chemotherapy can be curative for patients with early-stage OC, most women with advanced OC suffer repeated recurrences of the disease with a progressively shorter disease-free interval [2] and platinum resistance [3]. Unfortunately, there are few interventions that can postpone or stop the malignant course of the illness for patients who progressed after standard regimens. As no apparent breakthrough in the treatment of OC over the past two decades, the five-year overall survival rate is still <30% for patients with advanced-stage OC [4].

Angiogenesis plays an important role in the oncogenesis, development, and metastasis of OC [5]. Apatinib, as an inhibitor of vascular endothelial growth factor receptor 2, is widely used in China as an antiangiogenic drug. Studies have confirmed the antitumor activity of apatinib in the treatment of lung cancer [6], liver cancer [7], and other malignant tumors. For OC, increasing clinical studies have confirmed that apatinib alone or apatinib combined with chemotherapy can prolong the progression-free survival (PFS) of recurrent OC that is resistant to platinum-based chemotherapeutics [8–10]. However, due to the small sample size of relevant clinical studies and a lack of multicenter randomized controlled clinical trials, there is still a lack of sufficient evidence to confirm the clinical efficacy and safety of apatinib combined with chemotherapy in the treatment of OC. Therefore, we have performed a systematic review and meta-analysis to assess the efficacy and safety of apatinib plus chemotherapy.

## 2. Materials and Methods

### 2.1. Inclusion Criteria

The systematic review and meta-analysis were conducted according to the Preferred Reporting Items for Systematic Reviews and Meta-Analyses (PRISMA) guidelines. The inclusion criteria for the studies were as follows: (1) the type of included study was a randomized controlled trial (RCT) or case-control study (CCS), and there was no limit to follow-up procedures or type of setting; (2) the participants were confirmed to have OC pathologically or computed tomography, and there was no restriction on gender, race, or nationality for them; and (3) the intervention was apatinib combined with chemotherapy versus chemotherapy alone, and no patients had received chemotherapy, radiotherapy, targeted therapy, or other therapies within one month before treatment.

### 2.2. Exclusion Criteria

We excluded studies with any of the following characteristics: (1) the study was neither RCT nor CCS and involved nonovarian carcinoma, nonapatinib, or apatinib alone; (2) the patients were not confirmed to have OC; (3) the control measures did not include chemotherapy but other therapies such as operation or radiotherapy; (4) the data could not be extracted or were repeated; (5) the study was a review or unrelated meta-analyses, animal study, case report, conference abstracts, or letters to journal editors; and (6) studies had no data on tumor response, tumor-associated antigen (TAA), survival, and adverse drug reaction (ADR).

### 2.3. Retrieval Strategy

The retrieval strategy was built using MeSH and free words. The retrieval form was (apatinib OR rivoceranib esylate OR YN968D1 OR YN-968D1 OR rivoceranib OR apatinib mesylate) AND (“Ovarian Neoplasms” [Mesh] OR Neoplasm, Ovarian OR Ovarian Neoplasm OR Ovary Neoplasms OR Neoplasm, Ovary OR Neoplasms, Ovary OR Ovary Neoplasm OR Neoplasms, Ovarian OR Ovary Cancer OR Cancer, Ovary OR Cancers, Ovary OR Ovary Cancers OR Ovarian Cancer OR Cancer, Ovarian OR Cancers, Ovarian OR Ovarian Cancers OR Cancer of Ovary OR Cancer of the Ovary). Two investigators independently retrieved all related studies from the following databases: China Biological Medicine Database (CBM), China National Knowledge Infrastructure Database (CNKI), Chinese Scientific Journals Full-Text Database (VIP), Wanfang Data, Cochrane Library, PubMed, Greenmedical, and EBSCO (up to October 2020). In addition, we evaluated all related systematic reviews or meta-analyses and selected eligible studies from their references.

### 2.4. Study Selection

According to the predefined inclusion and exclusion criteria, two investigators independently selected eligible studies. Any disagreements were resolved by discussion between these two or with a third investigator.

### 2.5. Methodological Quality Assessment

The methodological quality of the included RCTs was assessed using the risk of bias tool of the Review Manager software 5.3. The criteria for assessment of the risk of bias included specific methods of randomization and allocation concealment, the blinding method, and reporting of dropouts or withdrawal of patients and other bias according to the Cochrane Evaluation Handbook for Systematic Reviews of Interventions Version 5.3. We judged each item as “yes” for a low risk of bias, “no” for high risk, and “unclear” otherwise. Two investigators independently assessed the risk of methodological bias of all included studies. Any disagreements about the decisions of high, low, or unclear risk were resolved by discussion with each other or a third investigator. Similarly, two investigators independently evaluated the quality of the included CCSs by Newcastle–Ottawa scale (NOS). Any disagreements about the decisions of the score were resolved by discussion with each other or with a third investigator.

### 2.6. Outcome Definition

Primary outcomes were tumor response, TAA, and survival. The outcomes for tumor response were the objective response rate (ORR) and disease control rate (DCR) according to the World Health Organization (WHO) guidelines for solid tumor responses or Response Evaluation Criteria in Solid Tumors (RECIST) [11]. The indicators used were complete response (CR), partial response (PR), no change (NC), and progressive disease (PD). The ORR was equal to CR plus PR, and the DCR was equal to CR plus PR and NC. The survival was assessed using overall survival (OS), time to progression (TTP), and PFS. Secondary outcomes were ADRs, which were identified according to the WHO standards [12] or Common Terminology Criteria for Adverse Events (CTCAE) [13].

### 2.7. Data Extraction

Three researchers independently extracted the following information from the included RCTs and CCSs: the first author's name, time of publication, treatment process, age, gender, sample size, dose of apatinib, chemotherapy regimen, and outcomes including ORR, DCR, OS, TTP, PFS, TAA, and ADRs. For crossover studies, only data from the first portion of the study would have been incorporated to avoid possible carryover effects of medications into the second part of the study and make these studies more comparable to those studies not of crossover design. If the reports were sufficiently detailed, the data were extracted directly from the papers. Otherwise, we contacted the authors for further information. When no author replied, the data were reconstructed using a software graph digitizer scout according to the graphed data in the paper [14, 15].

### 2.8. Statistical Analysis

Meta-analysis was implemented by two reviewers using Review Manager 5.3 (as recommended by the Cochrane Collaboration). The odds ratios (OR) and 95% confidence interval (CI) were calculated, and *P* < 0.05 was considered statistically significant. Statistical heterogeneity of the results among the studies was assessed by Cochran's *χ*^2^ test and the *I*^2^ statistic, and the inconsistency was calculated by *I*^2^. When *I*^2^ ≤ 50% and *P* > 0.1, there was no statistical heterogeneity among studies; if *I*^2^ > 50% and *P* < 0.1, there was statistical heterogeneity among studies. When heterogeneity was confirmed, the random-effects model (REM) was used. Otherwise, a fixed-effects model (FEM) was used. If *I*^2^ > 50% and with good clinical consistency, we adopted a random-effects model (REM). Otherwise, we discarded the outcome. Subgroup analyses were performed according to apatinib alone or plus different chemotherapy therapies and revealed their influence on the clinical effect of apatinib combined with chemotherapy. Furthermore, publication bias was assessed by funnel plot symmetry, the Egger test, and the Begg test with *P* < 0.05 suggesting obvious publication bias [16].

According to recent guidance [17], we established a subgroup analysis model to examine heterogeneity. Moreover, subgroup analysis was performed according to study type, study sample size, treatment process, dose of apatinib, chemotherapy regimen, and cycle aimed to reveal their influence on the clinical efficacy of apatinib combined with chemotherapy. We conducted a univariate metaregression for the relationship between each variable and tumor response. We also conducted multiple regression analysis, adjusting for the OR of tumor response at baseline, dose of apatinib, and different chemotherapy regimens.

We chose the STATA 11.0 software (STATA Corporation, College Station, TX) as a graphical tool to present the results' robustness by removing the trials one by one.

## 3. Results

### 3.1. Search Results

**Figure 1:**
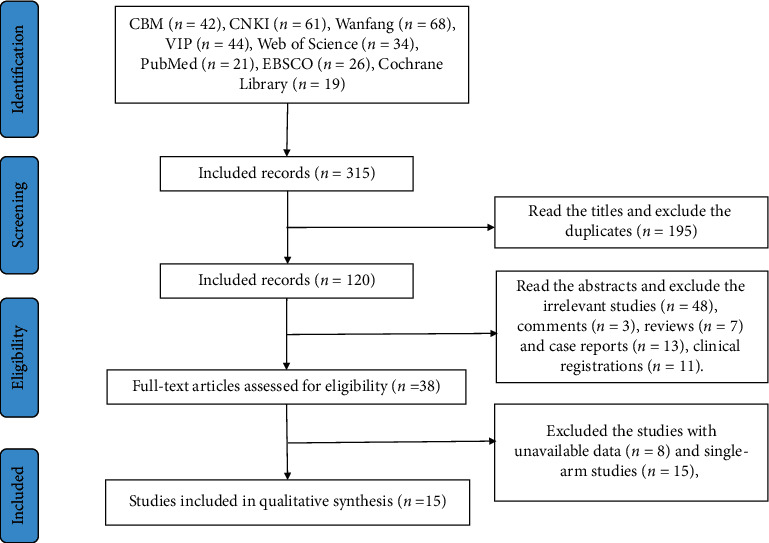
Articles retrieved and assessed for eligibility.

Following the process of the PRISMA flow diagram in Figure 1, 315 potentially relevant publications were identified. All records were imported into EndNote X6. Two reviewers screened all records using a three-step process. First, we screened the titles, excluding the duplicate including 195 records. Second, we read the abstracts and excluded irrelevant studies (*n* = 48), comments (*n* = 3), reviews (*n* = 7), case reports (*n* = 13), and clinical registrations (*n* = 11). Third, we assessed the full texts and excluded studies with unavailable data (*n* = 8) and single-arm studies (*n* = 15). Finally, we included 15 studies including 7 CCSs and 8 RCTs (Figure 1 and Table 1).

### 3.2. Characteristics of the Included Trials

This meta-analysis involved 15 studies containing 1,020 ovarian carcinoma patients who progressed after standard regimens in China. Combined administration with apatinib and chemotherapy was administered in 504 cases, and 516 cases were administered chemotherapy alone. Detailed information of the 15 studies is presented in Table 1.

### 3.3. Methodological Quality Assessment

**Figure 2:**
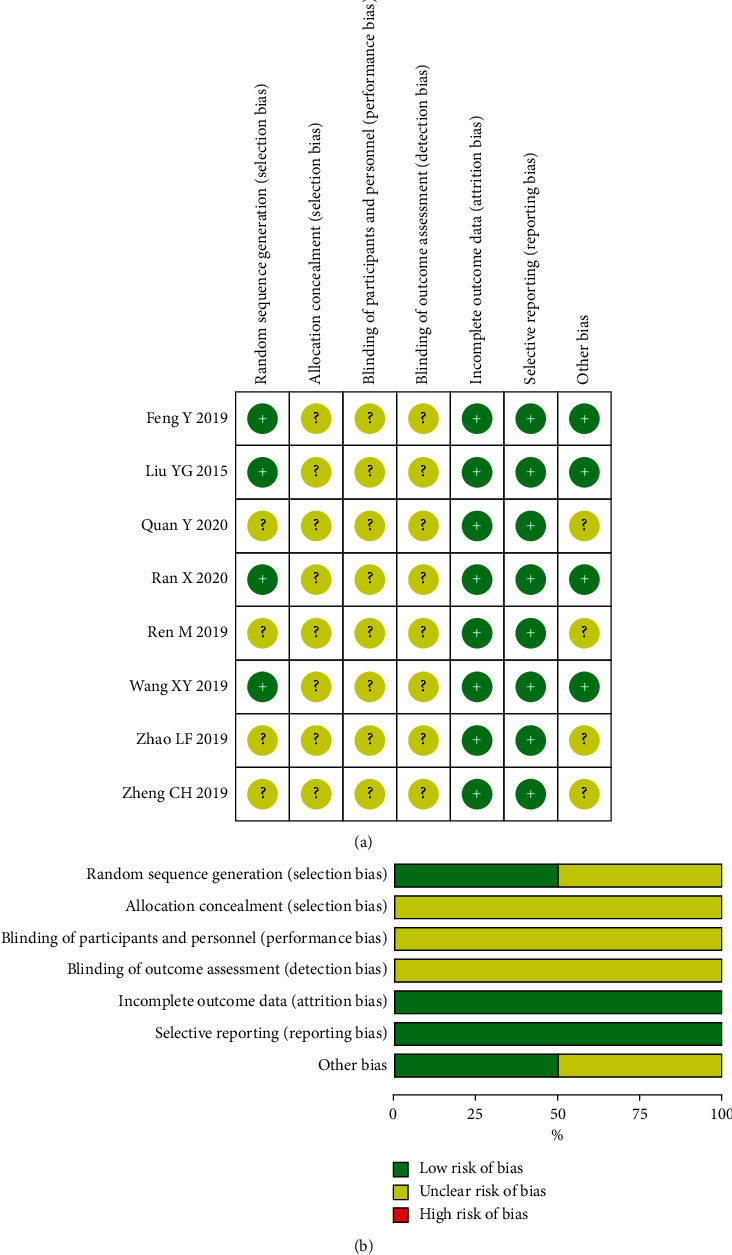
The risk of methodological bias: (a) risk of bias summary and (b) risk of bias graph.

The included RCTs underwent a quality assessment using the risk of bias tool of the Review Manager software 5.3, and the outcome is shown in Figure 2. The quality of the included case-control studies was assessed by NOS, and the outcome is shown in Table 2.

### 3.4. Objective Response Rate

**Figure 3:**
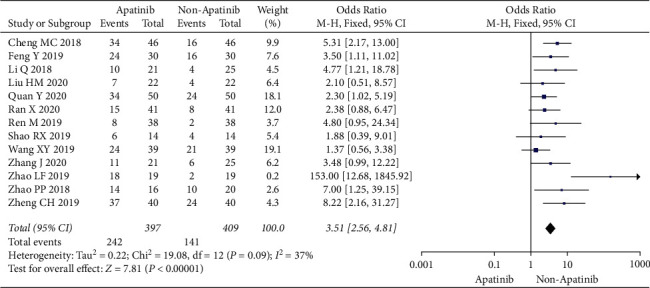
Objective response rate of chemotherapy plus apatinib in comparison with chemotherapy alone in the patients with ovarian carcinoma.

Cochran's *χ*^2^ test showed statistical heterogeneity for ORR (*P*=0.09; *I*^2^ = 15%). Therefore, we synthesized the data using an REM. Compared with chemotherapy alone, the results of the meta-analysis showed that apatinib in combination with chemotherapy significantly increased ORR (OR = 3.55; 95% CI 2.31 to 5.47; *P* < 0.00001; Figure 3).

### 3.5. Disease Control Rate

**Figure 4:**
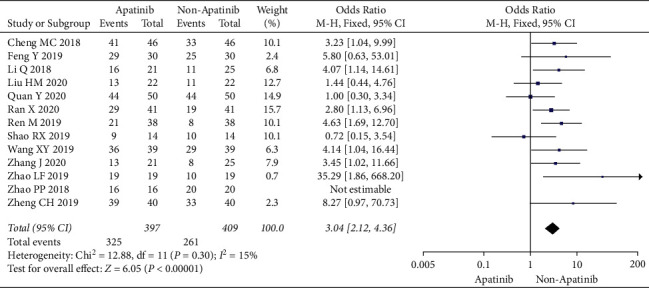
Disease control rate of chemotherapy plus apatinib in comparison with chemotherapy alone in the patients with ovarian carcinoma.

Cochran's *χ*^2^ test and *I*^2^ statistic showed minimal heterogeneity for DCR (*P*=0.30; *I*^2^ = 15%). Therefore, we synthesized the data using an FEM. Compared with chemotherapy alone, the results of the meta-analysis showed that apatinib plus chemotherapy significantly increased DCR (OR = 3.04; 95% CI 2.12 to 4.36; *P* < 0.00001; Figure 4).

### 3.6. Overall Survival

**Figure 5:**
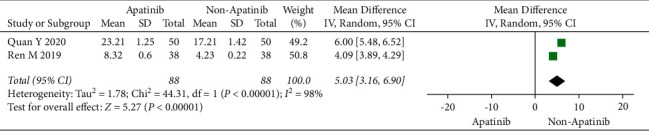
Overall survival of chemotherapy plus apatinib in comparison with chemotherapy alone in the patients with ovarian carcinoma.

Cochran's *χ*^2^ test and *I*^2^ statistic showed obvious heterogeneity for OS (*P* < 0.00001; *I*^2^ = 98%). Therefore, we synthesized the data using an REM. The results demonstrated that apatinib plus chemotherapy significantly increased OS (OR = 5.03; 95% CI 3.16 to 6.90; *P* < 0.00001; Figure 5).

### 3.7. Levels of Tumor-Associated Antigen

**Figure 6:**
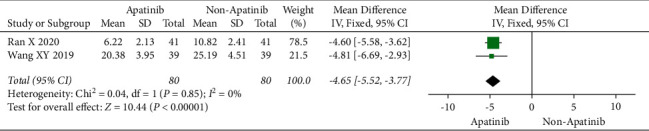
The level of CEA chemotherapy plus apatinib in comparison with chemotherapy alone in the patients with ovarian carcinoma.

**Figure 7:**
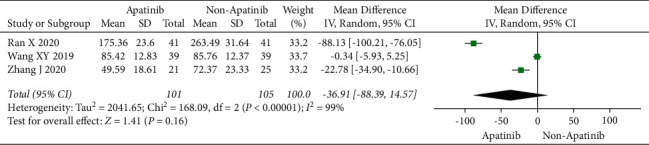
The level of CA125 of chemotherapy plus apatinib in comparison with chemotherapy alone in the patients with ovarian carcinoma.

Cochran's *χ*^2^ test and *I*^2^ statistics showed statistical heterogeneity for CA125 (*P* < 0.00001; *I*^2^ = 98%) and no heterogeneity for CEA (*P*=0.85; *I*^2^ = 0%). Thus, we synthesized the data of CA125 and CEA using REM and FEM, respectively. The results of the meta-analysis demonstrated that apatinib in combination with chemotherapy significantly decreased the level of CEA (MD = −4.65; 95% CI −5.52 to −3.77; *P* < 0.00001; Figure 6), although did not significantly decreased the level of CA125 (MD = −36.91; 95% CI −88.39 to 14.57; *P*=0.16; Figure 7).

### 3.8. Adverse Drug Reactions

Cochran's *χ*^2^ test and *I*^2^ statistics showed statistical heterogeneity for hypertension (*P*=0.02; *I*^2^ = 53%) and hand-foot syndrome (*P*=0.04; *I*^2^ = 55%); minimal heterogeneity for proteinuria (*I*^2^ = 30%); and no heterogeneity (*P* > 0.1; *I*^2^ = 0%) for myelosuppression, leucopenia, gastrointestinal reaction, nausea/vomiting, liver/renal dysfunction, and fatigue. Therefore, we synthesized the OR of hypertension and hand-foot syndrome using an REM and of other ADRs data using an FEM. The results of the meta-analysis demonstrated that apatinib in combination with chemotherapy resulted in a higher risk of hypertension (OR = 3.18; 95% CI 1.63 to 6.20; *P* < 0.001), proteinuria (OR = 3.14; 95% CI 1.51 to 6.52; *P*=0.002), and hand-foot syndrome (OR = 4.39; 95% CI 1.59 to 12.15; *P*=0.004). No statistical differences were shown in myelosuppression, leucopenia, gastrointestinal reaction, nausea/vomiting, liver/renal dysfunction, and fatigue between the two groups (Table 3 and Figures S1–S9).

### 3.9. Subgroup and Metaregression Analysis

Subgroup analyses of study type, dosage of apatinib, study sample size, treatment process, dosage of apatinib, chemotherapy regimen, and cycle between treatment and control groups were performed (Table 4 and Figures S10–S21).

#### 3.9.1. Study Type

Randomized clinical trials and case-control studies: subgroup analysis demonstrated that the heterogeneity (*I*^2^ = 62%; *P*=0.01) of RCTs in ORR was significant. Multiple metaregression analysis also found statistical significance in the correlation between the study type and DCR (*P*=0.043). Therefore, the heterogeneity of the studies in ORR and DCR might be from the RCTs.

#### 3.9.2. Study Sample Size

Fifty patients or more versus less than 50 patients: subgroup analysis demonstrated that there was a significant difference between the subgroups with different sample sizes in heterogeneity. Univariate metaregression analysis found statistical significance in the correlation between the study sample size and ORR (*P*=0.029). Additionally, univariate and multiple metaregression both found statistical significance in the correlation between the study sample size and DCR (*P*=0.018 and 0.030, respectively). Thus, the study sample size might be the other source of the heterogeneity of ORR and DCR.

#### 3.9.3. Treatment Process

Failure of first-line chemotherapy (FFC), failure of platinum-based chemotherapy (FPC), and failure of second- or multiple-line chemotherapy (FSMC): subgroup analysis demonstrated that the heterogeneity (*I* = 55%; *P*=0.07) of the subgroup with patients suffering FSMC was more obvious than other subgroups in ORR, indicating the subgroup might increase the overall heterogeneity in ORR. Additionally, subgroup analyses showed that patients receiving apatinib plus chemotherapy who suffered FSMC had significantly better ORR (OR = 6.00; 95% CI 2.31 to 15.58; *P* < 0.001) and DCR (OR = 3.75; 95% CI 2.19 to 6.42; *P* < 0.001).

#### 3.9.4. Dosage of Apatinib

A dosage of 250, 425, 500, and unclear mg/d. Subgroup analysis demonstrated that patients receiving apatinib plus chemotherapy who were administrated with apatinib of 500 mg/d had significantly better ORR (OR = 4.10; 95% CI 2.10, 8.00; *P* < 0.001) and DCR (OR = 3.08; 95% CI 1.98, 4.81; *P* < 0.001).

#### 3.9.5. Chemotherapy Regimen

Platinum, taxane or anthracycline, taxane + platinum, gemcitabine, and taxane + anthracycline: subgroup analysis demonstrated that the heterogeneity of the subgroup with patients suffering chemotherapy based on taxane or anthracycline was obvious in ORR (*I*^2^ = 58%; *P*=0.04) and DCR (*I*^2^ = 43%; *P*=0.13), indicating the subgroup might increase the overall heterogeneity in ORR and DCR. Additionally, the heterogeneity of the subgroup with patients suffering chemotherapy based on taxane plus platinum was more obvious than those of other subgroups in DCR (*I*^2^ = 62%; *P*=0.10), indicating the subgroup might also increase the overall heterogeneity in DCR. Subgroup analyses also showed that patients receiving apatinib plus chemotherapy who suffered chemotherapy based on taxane plus anthracycline had significantly better ORR (OR = 8.22; 95% CI 2.16, 31.27; *P*=0.002), and patients receiving apatinib plus platinum-based chemotherapy had considerable DCR (OR = 4.85; 95% CI 1.94, 12.16; *P* < 0.001).

#### 3.9.6. Chemotherapy Cycle

Less than 4 cycles, 4–6 cycles, more than 6 cycles, and unclear cycles. Subgroup analysis demonstrated that the heterogeneity of the subgroup with patients receiving unclear chemotherapy cycles was more obvious than other subgroups in ORR (*I*^2^ = 55%; *P*=0.05) and DCR (*I*^2^ = 51%; *P*=0.07), indicating that the subgroup might increase the overall heterogeneity in ORR and DCR. Additionally, subgroup analyses showed that patients receiving apatinib plus chemotherapy who received more than 6 chemotherapy cycles had significantly better ORR (OR = 8.22; 95% CI 2.16, 31.27; *P*=0.002), and patients received apatinib plus chemotherapy who receiving 4–6 chemotherapy cycles had remarkable DCR (OR = 3.84; 95% CI 1.75, 8.45; *P* < 0.001).

### 3.10. Publication Bias Analysis

According to the results of funnel plots and Egger/Begg tests, publication bias was shown in these trials for ORR (*P*=0.017; 95% CI 0.33, 2.67), DCR (*P*=0.013; 95% CI 0.50, 3.36), and hand-foot syndrome (*P* < 0.01; 95% CI −2.53, −1.65), and the other results had no apparent publication bias (Table S1 and Figures S22–S32).

### 3.11. Sensitivity Analysis

Sensitivity analysis was conducted for ORR, DCR, CA125, myelosuppression, hypertension, proteinuria, leucopenia, gastrointestinal reaction, nausea/vomiting, hand-foot syndrome, and liver/renal dysfunction by removing the trials one by one. The results of sensitivity analysis demonstrated that except for CA125, most results had good robustness before and after removing trials (Figures S33–S43).

## 4. Discussion

Although OC can be cured in the early stages, once it progresses to an advanced or late stage, few interventions can postpone or stop the malignant illness leading to death. According to the European Society for Medical Oncology and National Comprehensive Cancer Network (NCCN) guidelines for OC [33, 34], chemotherapy regimens based on carboplatin plus paclitaxel are the standard treatments for patients with stage II–IV OC. However, many patients relapse after standard treatment and likely develop platinum resistance [35]. There are many available second-line treatment regimens including liposomal doxorubicin, paclitaxel, gemcitabine, etoposide, and so on. Nevertheless, the curative effect of these regimens is extremely limited, of which the objective response rate is less than 30% and the median progression-free survival (PFS) time is 3 to 4 months [36, 37]. As no appropriate regimens were available for patients after the second-line treatment regimen [38], OC can easily lead to death once second-line treatment failed.

Angiogenesis has been proved to play an important role in the relapse and metastasis of many cancers such as OC [39] and breast cancer [40]. Vascular endothelial growth factor (VEGF) and its receptor (VEGFR) have been recognized as the critical role of the process of angiogenesis [41]. Over the past decades, many therapeutic agents have been developed targeting to inhibit VEGF and VEGFR, such as bevacizumab and panitumumab [42, 43]. Apatinib, a small molecule inhibitor, can suppress the migration and proliferation of endothelial cells motivated via reducing the tyrosine kinase activity of VEGFR-2 [44]. Ding et al. [45] found that apatinib could suppress tumor growth, migration, and epithelial-mesenchymal transition of OC cells in vivo and in vitro by inhibiting the JAK/STAT3, PI3K/AKT, and Notch signal pathways. Moreover, apatinib has also been proved to promote ROS-dependent apoptosis as well as autophagy in OC cells [46] and inhibit the glycolysis of OC cells via suppressing the VEGFR2/AKT1/SOX5/GLUT4 [47]. Furthermore, apatinib can sensitize resistant tumor cells to chemotherapy drugs and increase the effectiveness of conventional chemotherapy drugs. Tong et al. [48] demonstrated that apatinib could reverse the resistance to docetaxel, daunorubicin, and vincristine in K562/ADR cells. Apatinib has been also proved to improve the antitumor effects of paclitaxel (albumin binding type) in platinum-resistant ovarian cancer cell line and xenograft models [49]. In the past decades, there have been increasing clinical studies on the treatment of OC administrated with apatinib. Chen et al. [50] included 117 eligible patients to evaluate the efficacy and safety of apatinib with a low dose of 250 mg/d in the treatment of platinum-resistant or platinum-refractory OC patients and then found that the patients administrated with apatinib had satisfactory ORR, DCR, and OS. Miao et al. [51] carried out a phase II study of apatinib and advocated that apatinib 500 mg daily PO is a feasible treatment in patients with recurrent, platinum-resistant, pretreated epithelial ovarian cancer. Additionally, studies have proved that the combination of apatinib and oral etoposide showed promising efficacy and a manageable toxicity profile to treat platinum-resistant or platinum-refractory ovarian cancer [10, 52]. A real-world study also verified apatinib produced moderate improvements in progression-free survival in patients with platinum-resistant epithelial OC both as maintenance therapy following chemotherapy and as single-agent salvage therapy, and apatinib may be effective for women with platinum-resistant recurrent epithelial ovarian cancer [8].

However, there has been not enough evidence to prove the efficacy and safety of apatinib combined with chemotherapy in the treatment of ovarian carcinoma. To evaluate whether and how the administration of apatinib plus chemotherapy improves clinical efficacy and its safety in patients with ovarian carcinoma who progressed after standard regimens, we included 8 RCTs and 7 CCSs for meta-analysis. Overall, the methodological quality of 8 RCTs and 7 CCSs was found to be medium and high, respectively. This meta-analysis proved that apatinib combined with chemotherapy significantly improved ORR, DCR, and OS and statistically decreased the level of CEA. In relation to adverse drug reactions, apatinib plus chemotherapy increased incidence of hypertension, hand-foot syndrome, and proteinuria while having a similar risk of myelosuppression, leucopenia, gastrointestinal reaction, nausea/vomiting, liver/renal dysfunction, and fatigue when compared to administration with chemotherapy alone. Subgroup and metaregression analysis showed the difference of study type, study sample size, treatment process, dosage of apatinib, chemotherapy regimen, and cycle might increase the overall heterogeneity in ORR and DCR, and some differences were seen between treatment process, dosage of apatinib, chemotherapy regimen, and cycle. Nevertheless, there was slight or no difference among the other subgroups. Publication bias was only shown in these trials for ORR, DCR, and hand-foot syndrome. The results of sensitivity analysis demonstrated that most results had good robustness before and after removing trials one by one.

This meta-analysis has several limitations. Firstly, all studies included in our meta-analysis were published in Chinese. We used a comprehensive search strategy and tried to reduce selection bias by searching Cochrane Library, PubMed, Greenmedical, and EBSCO. However, no studies published in English were found. Although we included 15 studies covering 1,020 participants, only two studies included no less than 100 patients. More large-scale randomized double-blind control trials are needed to overcome methodological and reporting flaws. In addition, the methods of blinding of all studies were not reported. Most trials had unclear bias risk. Except for the correlation between the study sample size and DCR, univariate and multiple metaregression analysis did not find other positive correlations. In general, all of these limitations might have resulted in an insufficient evaluation of the outcomes.

## 5. Conclusion

In conclusion, most of the included studies showed an unclear risk of bias, and the methodological quality of them was found to be medium and high. Compared with chemotherapy alone, apatinib plus chemotherapy significantly increased ORR, DCR, and OS, with the acceptable risk of adverse drug action. For patients suffering the failure of no less than second-line chemotherapy, 500 mg/day apatinib plus chemotherapy (especially based on taxanes plus anthracycline or platinum for more than 4 cycles) might be the optimal regimen aimed to produce the desired tumor response. Thus, apatinib plus chemotherapy was more effective than chemotherapy alone for the treatment of ovarian carcinoma who progressed after standard regimens. Chemotherapy plus apatinib may provide an additional option for the treatment of suitable patients with the failure of standard regimens. In addition, can apatinib plus chemotherapy improve the long-time survival rate? Can apatinib improve clinical efficacy for drug-resistant patients? Which is the optimal dosage of apatinib and chemotherapy regimen to achieve the best antitumor effect? All these questions need adequately powered and high-quality randomized clinical trials with short- and long-term follow-ups in the future.

## Figures and Tables

**Table 1:** Characteristics of the included trials.

Trial type	First author, year	Patient			Apatinib (mg·d^−1^)	Intervention (regimen, cycle, or duration)	Criteria	Outcomes
		Age (years)	No. (E/C)	TP				
CCS	Li, 2018 [18]	26–70^a^	21/25^d^	FSMC	500	E: apatinib + taxane or anthracycline, 6 cycles C: taxane or anthracycline, 6 cycles	RECIST 1.1, CTCAE	O1, 4
CCS	Cheng et al., 2018 [19]	Un	46/46^d^	FSMC	250	E: apatinib + taxanes, 6 cycles C: taxane, 6 cycles	Un, CTCAE	O1, 4
CCS	Shao et al., 2019 [20]	64.2 ± 1.63/58.55 ± 1.45^b^	54/54^d^	Un	500	E: apatinib + taxane + platinum, Un C: taxane + platinum, Un	Un, Un	O1. 4
CCS	Liu et al., 2020 [21]	36.9 ± 5.1/45.1 ± 4.9^b^	22/22^d^	FSMC	500	E: apatinib + taxanes or anthracycline, Un C: taxanes or anthracycline, Un	RECIST 1.1, WHO	O1, 4
CCS	Zhang and Shi, 2020 [22]	18–70^a^	21/25^d^	FPC	500	E: apatinib + gemcitabine, Un C: gemcitabine, Un	RECIST 1.1, Un	O1, 3, 4
CCS	Zhang and Xiong, 2019 [23]	53.4 ± 4.2/53.3 ± 4.6^b^	30/30^d^	Un	500	E: apatinib + platinum, 8 cycles C: platinum, 8 cycles	Un	O4
CCS	Zhao, 2018 [24]	49–75^a^	16/20^d^	FPC	500	E: apatinib + anthracycline, 6 cycles C: anthracycline, 6 cycles	RECIST 1.1, Un	O1, 4
RCT	Feng et al., 2019 [25]	45.8 ± 5.7/46.2 ± 6.1^b^	30/30^d^	FPC	Un	E: apatinib + platinum, 4 cycles C: platinum, 4 cycles	Un, Un	O1, 4
RCT	Ren, 2019 [26]	74.0 ± 1.3/75.0 ± 1.5^b^	38/38^d^	FSMC	500	E: apatinib + platinum, Un C: platinum, Un	WHO, Un	O1, 2, 3, 4
RCT	Wang and Qu, 2019 [27]	50.68 ± 16.84^C^	39/39^d^	FPC	425	E: apatinib + taxane + platinum, 3 cycles C: taxane + platinum, 3 cycles	RECIST 1.1, Un	O1, 3, 4
RCT	Liu, 2015 [28]	48.5 ± 5.5/48.0 ± 5.0^b^	37/37^d^	Un	850	E: apatinib + taxane + platinum, 4 cycles C: taxane + platinum, 4 cycles	WHO	O4
RCT	Zhao et al., 2019 [29]	54.29 ± 6.87/54.76 ± 6.72^b^	19/19^d^	FSMC	500	E: apatinib + taxane or anthracycline, Un C: taxane or anthracycline, Un	Un	O1
RCT	Zheng et al., 2019 [30]	60.5 ± 5.1/60.1 ± 4.9^b^	40/40^d^	FPC	Un	E: apatinib + taxane + anthracycline, 6–8cycles C: taxane + anthracycline, 6–8cycles	WHO, Un	O1, 4
RCT	Quan, 2020 [31]	55.89 ± 7.76/55.21 ± 7.71^b^	50/50^d^	FPC	250	E: apatinib + taxane or anthracycline, Un C: taxane or anthracycline, Un	Un, Un	O1, 2, 4
RCT	Ran and Liu, 2020 [32]	52.10 ± 12.19/51.37 ± 12.53^b^	41/41^d^	FFC	500	E: apatinib + gemcitabine, 3 cycles C: gemcitabine, 3 cycles	RECIST 1.1, CTCAE	O1, 2, 3, 4

Note: CCS: case-control study; RCT: randomized clinical trial; E: experimental group; C: control group; Un: unclear; ^a^all patients (range); ^b^E/C (mean ± SD); ^C^all patients (mean ± SD); ^d^the number of patients in experimental and control groups; TP: treatment process; FFC: failure of first-line chemotherapy; FPC: failure of platinum-based chemotherapy; FSMC: failure of second-line or multiple-line chemotherapy; WHO: World Health Organization guidelines for solid tumor responses, adverse events, and quality of life; RECIST 1.1: Version 1.1 of Response Evaluation Criteria in Solid Tumors; CTCAE: Common Terminology Criteria for Adverse Events; O: outcomes; O1: tumor response including objective response rate and disease control rate; O2: survival status including overall survival, time to progression, and progression-free survival; O3: tumor-associated antigen; and O4: adverse drug reactions.

**Table 2:** Results of quality assessment using the Newcastle–Ottawa scale for case-control studies.

First author, year	Selection				Comparability of cases and controls on the basis of the design or analysis	Exposure			
	Adequate definition of cases	Representativeness of the cases	Selection of controls	Definition of controls		Ascertainment of exposure	Same method of ascertainment for cases and controls	Nonresponse rate	Scores
Li, 2018 [18]	☆	☆		☆	☆☆	☆	☆	☆	8
Cheng et al., 2018 [19]	☆	☆		☆	☆☆	☆	☆	☆	8
Shao et al., 2019 [20]	☆	☆		☆	☆☆	☆	☆	☆	8
Liu et al., 2020 [21]	☆	☆		☆	☆☆	☆	☆	☆	8
Zhang and Shi, 2020 [22]	☆	☆		☆	☆☆	☆	☆	☆	8
Zhang and Xiong, 2019 [23]	☆	☆		☆	☆☆	☆	☆	☆	8
Zhao, 2018 [24]	☆	☆		☆	☆☆	☆	☆	☆	8

☆: study can be awarded a maximum of one star for each numbered item within the selection and exposure categories; ☆☆: a maximum of two stars can be given for comparability.

**Table 3:** Meta-analysis results of adverse drug reactions (Figures S1–S9).

Outcomes	Number of trials	Experimental group (events/total)	Control group (events/total)	SM	OR (95% CI)	*I* ^2^	Heterogeneity	*P* value
Myelosuppression (Figure S1)	5	38/162	36/166	FEM	1.16 (0.61, 2.17)	0%	0.92	0.65
Leucopenia (Figure S2)	5	49/119	54/127	FEM	1.00 (0.56, 1.79)	0%	0.68	0.99
Hypertension (Figure S3)	11	98/339	40/351	REM	3.18 (1.63, 6.20)	53%	0.02	<0.001
Proteinuria (Figure S4)	6	28/148	12/160	FEM	3.14 (1.51, 6.52)	30%	0.21	0.002
Gastrointestinal reaction (Figure S5)	5	80/165	90/173	FEM	0.87 (0.55, 1.37)	0%	0.98	0.54
Nausea/vomiting (Figure S6)	5	40/161	40/161	FEM	1.00 (0.58, 1.73)	0%	0.78	1.00
Hand-foot syndrome (Figure S7)	7	60/201	19/213	REM	4.39 (1.59, 12.15)	55%	0.04	0.004
Liver/renal dysfunction (Figure S8)	4	32/113	38/117	FEM	0.81 (0.46, 1.44)	0%	0.74	0.47
Fatigue (Figure S9)	2	8/54	7/58	FEM	1.23 (0.41, 3.66)	0%	0.48	0.71

Note: SM: statistical method, REM: random-effects model, FEM: fixed-effects model, OR: odds ratio, and CI: confidence interval.

**Table 4:** Subgroup and metaregression analysis of ORR and DCR.

Subgroups	No. of studies	ORR						DCR					
		OR (95% CI)	*I* ^2^	Heterogeneity *P* value	*P* value	MM *P* value	UM *P* value	OR (95% CI)	*I* ^2^	Heterogeneity *P* value	*P* value	MM *P* value	UM *P* value
Subgroup and metaregression analysis according to study type (Figures S10–S11)													
CCS	6	3.91 (2.32, 6.59)	0%	0.76	<0.001	0.277	0.104	2.38 (1.37, 4.11)	4%	0.39	0.002	0.043	0.945
RCT	7	3.79 (1.82, 7.88)	62%	0.01	<0.001			3.64 (2.25, 5.89)	15%	0.24	<0.001		

Subgroup and metaregression analysis according to study sample size (Figures S12–S13)													
<50	6	5.54 (2.54, 12.12)	46%	0.10	<0.001	0.123	0.029	3.45 (1.96, 6.07)	12%	0.34	<0.001	0.018	0.030
≥50	7	2.56 (1.69, 3.87)	0%	0.43	<0.001			2.78 (1.74, 4.44)	27%	0.22	<0.001		

Subgroup and metaregression analysis according to treatment process (Figures S14–S15)													
FSMC	5	6.00 (2.31, 15.58)	55%	0.07	<0.001	0.810	0.123	3.75 (2.19, 6.42)	19%	0.29	<0.001	0.284	0.107
FPC	6	2.98 (1.77, 5.02)	23%	0.26	<0.001			2.99 (1.59, 5.62)	14%	0.33	<0.001		
FFC	1	2.38 (0.88, 6.47)	—	—	0.09			2.80 (1.13, 6.96)	—	—	0.03		
Unclear	1	1.88 (0.39, 9.01)	—	—	0.43			0.72 (0.15, 3.54)	—	—	0.69		

Subgroup and metaregression analysis according to dosage of apatinib (Figures S16–S17)													
250 mg	2	3.42 (1.51, 1.76)	46%	0.18	0.003	0.477	0.910	1.90 (0.85, 4.24)	48%	0.16	0.12	0.446	0.713
425 mg	1	1.37 (0.56, 3.38)	—	—	0.49			4.14 (1.04, 16.44)	—	—	0.04		
500 mg	8	4.10 (2.10, 8.00)	40%	0.11	<0.001			3.08 (1.98, 4.81)	28%	0.22	<0.001		
Unclear	2	5.03 (2.11, 12.00)	0%	0.34	<0.001			7.03 (1.51, 32.70)	0%	0.82	0.01		

Subgroup and metaregression analysis according to chemotherapy regimen (Figures S18–S19)													
Platinum	2	3.89 (1.52, 9.92)	0%	0.75	0.004	0.852	0.696	4.85 (1.94, 12.16)	0%	0.86	<0.001	0.435	0.456
Taxanes or anthracycline	6	4.98 (2.22, 11.19)	58%	0.04	<0.001			2.64 (1.52, 4.59)	43%	0.13	<0.001		
Taxanes + platinum	2	1.48 (0.68, 3.24)	0%	0.73	0.32			2.03 (0.76, 5.43)	62%	0.10	0.16		
Gemcitabine	2	2.76 (1.26, 6.03)	0%	0.64	0.01			3.02 (1.46, 6.25)	0%	0.79	0.003		
Taxanes + anthracycline	1	8.22 (2.16, 31.27)	—	—	0.002			8.27 (0.97, 70.73)	—	—	0.05		

Subgroup and metaregression analysis according to chemotherapy cycle (Figures S20–S21)													
<4	2	1.76 (0.90, 3.43)	0%	0.42	0.10	0.290	0.472	3.18 (1.49, 6.78)	0%	0.64	0.003	0.334	0.294
4–6	4	4.82 (2.67, 8.69)	0%	0.91	<0.001			3.84 (1.75, 8.45)	0%	0.89	<0.001		
>6	1	8.22 (2.16, 31.27)	—	—	0.002			8.27 (0.97, 70.73)	—	—	0.05		
Unclear	6	3.81 (1.62, 8.98)	55%	0.05	0.002			2.49 (1.52, 4.10)	51%	0.07	<0.001		

Note: ORR: objective response rate, DCR: disease control rate, CI: confidence interval, UM: univariate metaregression, MM: multiple metaregression, CCS: case-control study, RCT: randomized clinical trial, FFC: failure of first-line chemotherapy, FPC: failure of platinum chemotherapy, and FSMC: failure of second- or multiple-line chemotherapy.

## Data Availability

The retrieval strategy was built using MeSH and free words. The retrieval form was (apatinib OR rivoceranib esylate OR YN968D1 OR YN-968D1 OR rivoceranib OR apatinib mesylate) AND (“Ovarian Neoplasms” [Mesh] OR Neoplasm, Ovarian OR Ovarian Neoplasm OR Ovary Neoplasms OR Neoplasm, Ovary OR Neoplasms, Ovary OR Ovary Neoplasm OR Neoplasms, Ovarian OR Ovary Cancer OR Cancer, Ovary OR Cancers, Ovary OR Ovary Cancers OR Ovarian Cancer OR Cancer, Ovarian OR Cancers, Ovarian OR Ovarian Cancers OR Cancer of Ovary OR Cancer of the Ovary). Two investigators independently retrieved all related studies from the following databases: China Biological Medicine Database (CBM), China National Knowledge Infrastructure Database (CNKI), Chinese Scientific Journals Full-Text Database (VIP), Wanfang Data, Cochrane Library, PubMed, Greenmedical, and EBSCO (up to October 2020).

## Supplementary Materials

Supplementary Materials Supplementary Material 1. Figures S1–S21: forest plots of subgroup and metaregression analysis; Supplementary Material 2. Table S1 and Figures S22–32: results of publication bias analysis; and Supplementary Material 3. Figures S33–43: results of sensitivity analysis.
