# Supplementary material for: A Comparison of Chemotherapy Used with and without Apatinib for Patients with Ovarian Carcinoma Who Progressed after Standard Regimens: A Systematic Review and Meta-Analysis
Source: Evid Based Complement Alternat Med. 2021 Nov 3;2021:2292907. doi: 10.1155/2021/2292907 (PMC8580656; doi:10.1155/2021/2292907)
Supplement: Supplementary Materials — Supplementary Material 1. Figures S1–S21: forest plots of subgroup and metaregression analysis; Supplementary Material 2. Table S1 and Figures S22–32: results of publication bias analysis; and Supplementary Material 3. Figures S33–43: results of sensitivity analysis. [file 2292907.f1.zip › 2292907.f1/TABLE S1 (1).pdf]

Table S1: Publication bias analysis.

Publication bias analysis of clinical efficacy				
Egger/Begg's tests	ORR (Figure S22)		DCR (Figure S23)	CA125 (Figure S24)
Egger's test (*P*, 95%CI)	*P* = 0.017, 95%CI[0.33, 2.67]		*P* = 0.013, 95%CI[0.50, 3.36]	*P* = 0.211, 95%CI[-82.41, 51.71]
Begg's test (*P*)	*P* = 0.059		*P* = 0.024	*P* = 0.296
Publication bias analysis of adverse drug reactions				
Egger/Begg's tests	Hypertension (Figure S25)	Proteinuria (Figure S26)	Myelosuppression (Figure S27)	Leucopenia (Figure S28)
Egger's test (*P*, 95%CI)	*P* = 0.823, 95%CI[-2.85, 2.82]	*P* = 0.441, 95%CI[-1.88, 3.55]	*P* = 0.884, 95%CI[-1.16, -1.29]	*P* = 0.419, 95%CI[-2.26, 1.24]
Begg's test (*P*)	*P* = 0.755	*P* = 0.707	*P* = 1.000	*P* = 0.806
Egger/Begg's tests	Gastrointestinal reaction (Figure S29)	Nausea/vomiting (Figure S30)	Hand-foot Syndrome (Figure S31)	Liver/Renal dysfunction (Figure S32)
Egger's test (*P*, 95%CI)	P = 0.662, 95%CI[-1.82, 2.48]	*P* = 0.312, 95%CI[-1.58, 0.65]	*P* = 0.00, 95%CI[-2.53, -1.65]	*P* = 0.964, 95%CI[-3.98, 4.08]
Begg's test (*P*)	*P* = 0.806	*P* = 0.452	*P* = 0.02	*P* = 0.734

Note: CI: confidence interval, ORR: objective response rate, DCR: disease control rate.
