# Supplementary figures and images for: A Comparison of Chemotherapy Used with and without Apatinib for Patients with Ovarian Carcinoma Who Progressed after Standard Regimens: A Systematic Review and Meta-Analysis
Source: Evid Based Complement Alternat Med. 2021 Nov 3;2021:2292907. doi: 10.1155/2021/2292907 (PMC8580656; doi:10.1155/2021/2292907)

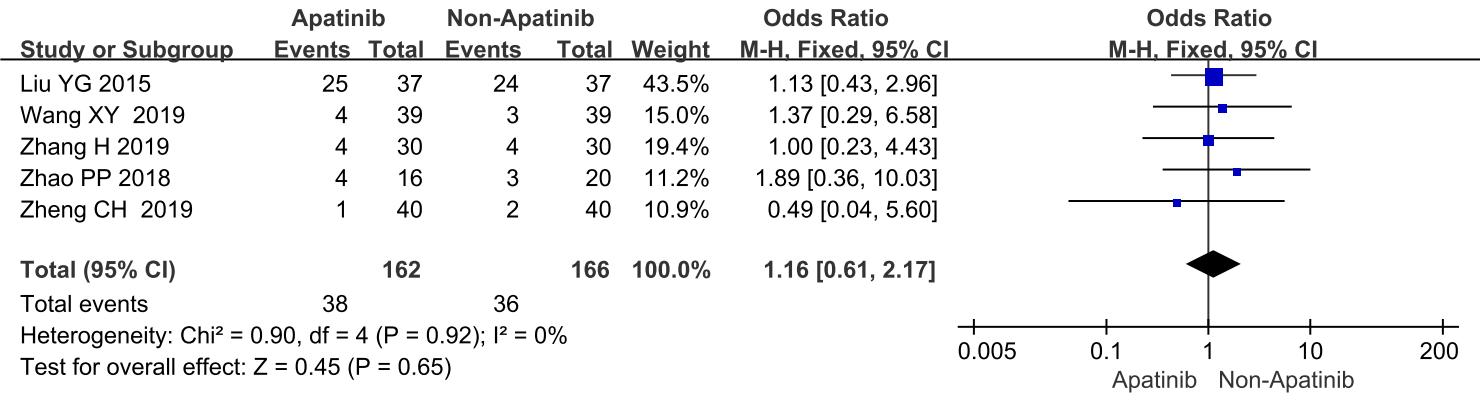

Supplement: Supplementary Materials — Supplementary Material 1. Figures S1–S21: forest plots of subgroup and metaregression analysis; Supplementary Material 2. Table S1 and Figures S22–32: results of publication bias analysis; and Supplementary Material 3. Figures S33–43: results of sensitivity analysis. [file 2292907.f1.zip › 2292907.f1/Figure S1 Meta-analysis results of myelosuppression.pdf]

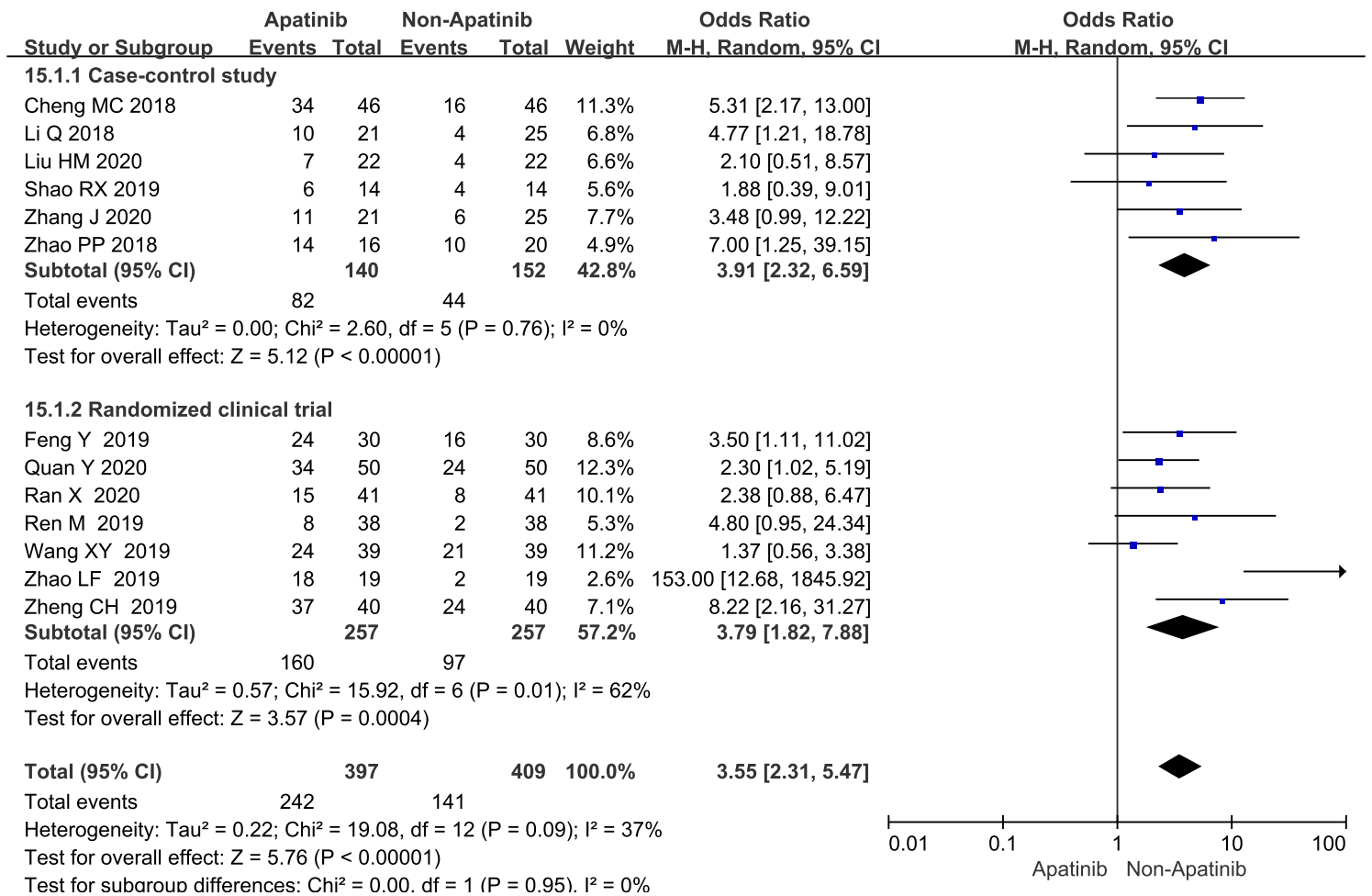

Supplement: Supplementary Materials — Supplementary Material 1. Figures S1–S21: forest plots of subgroup and metaregression analysis; Supplementary Material 2. Table S1 and Figures S22–32: results of publication bias analysis; and Supplementary Material 3. Figures S33–43: results of sensitivity analysis. [file 2292907.f1.zip › 2292907.f1/Figure S10 Subgroup analysis of ORR according to study type.pdf]

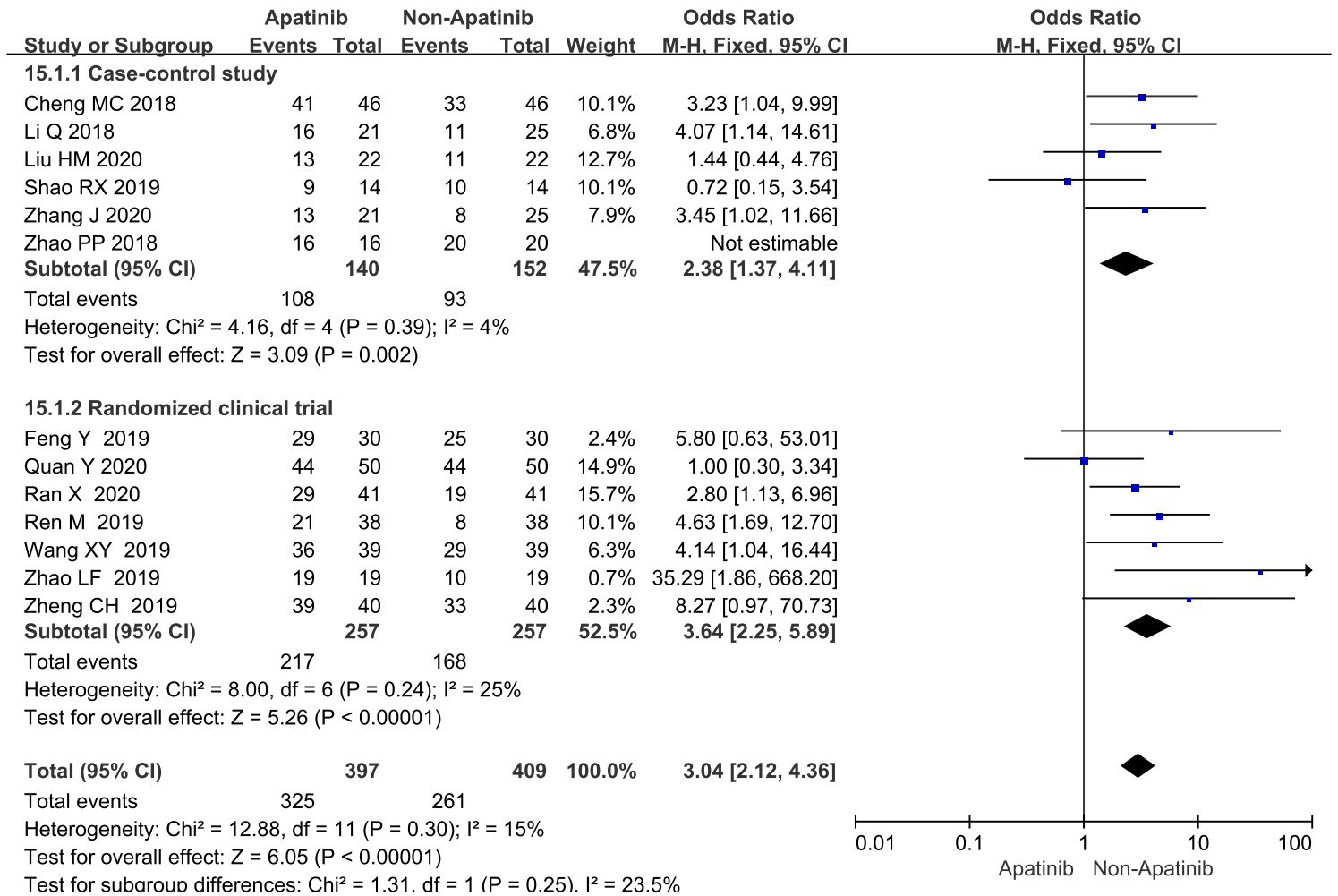

Supplement: Supplementary Materials — Supplementary Material 1. Figures S1–S21: forest plots of subgroup and metaregression analysis; Supplementary Material 2. Table S1 and Figures S22–32: results of publication bias analysis; and Supplementary Material 3. Figures S33–43: results of sensitivity analysis. [file 2292907.f1.zip › 2292907.f1/Figure S11 Subgroup analysis of DCR according to study type.pdf]

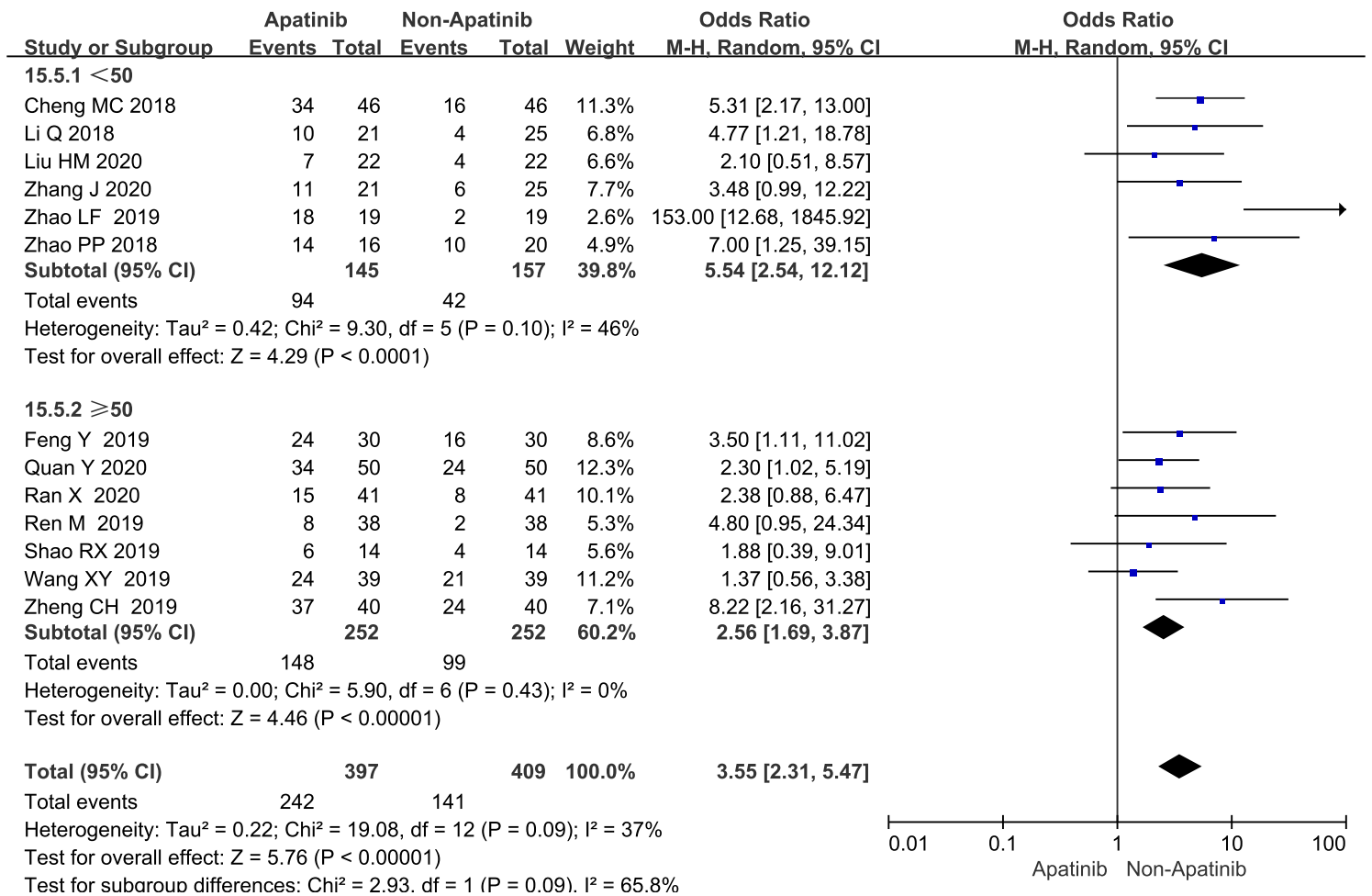

Supplement: Supplementary Materials — Supplementary Material 1. Figures S1–S21: forest plots of subgroup and metaregression analysis; Supplementary Material 2. Table S1 and Figures S22–32: results of publication bias analysis; and Supplementary Material 3. Figures S33–43: results of sensitivity analysis. [file 2292907.f1.zip › 2292907.f1/Figure S12 Subgroup analysis of ORR according to study sample size.pdf]

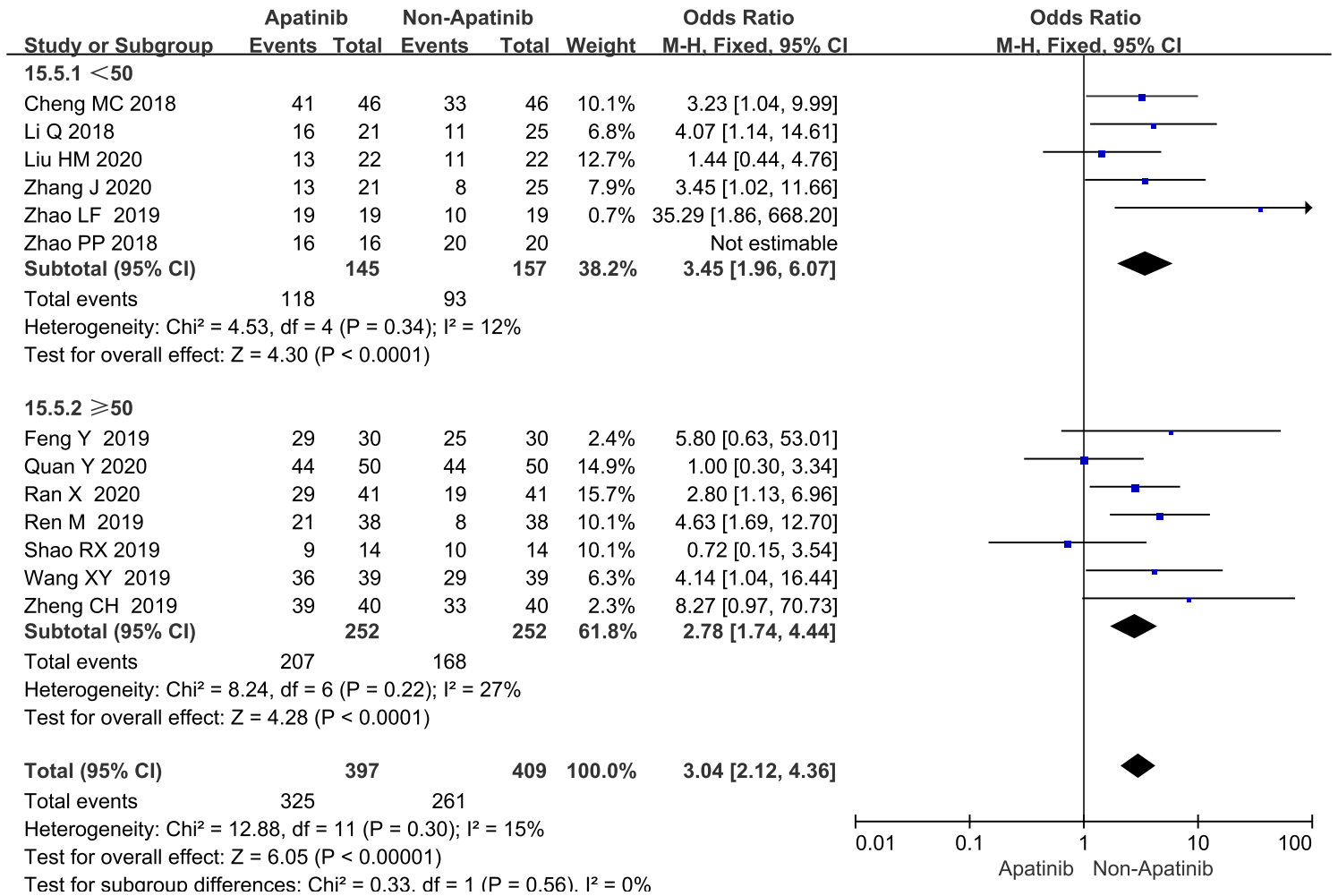

Supplement: Supplementary Materials — Supplementary Material 1. Figures S1–S21: forest plots of subgroup and metaregression analysis; Supplementary Material 2. Table S1 and Figures S22–32: results of publication bias analysis; and Supplementary Material 3. Figures S33–43: results of sensitivity analysis. [file 2292907.f1.zip › 2292907.f1/Figure S13 Subgroup analysis DCR according to study sample size.pdf]

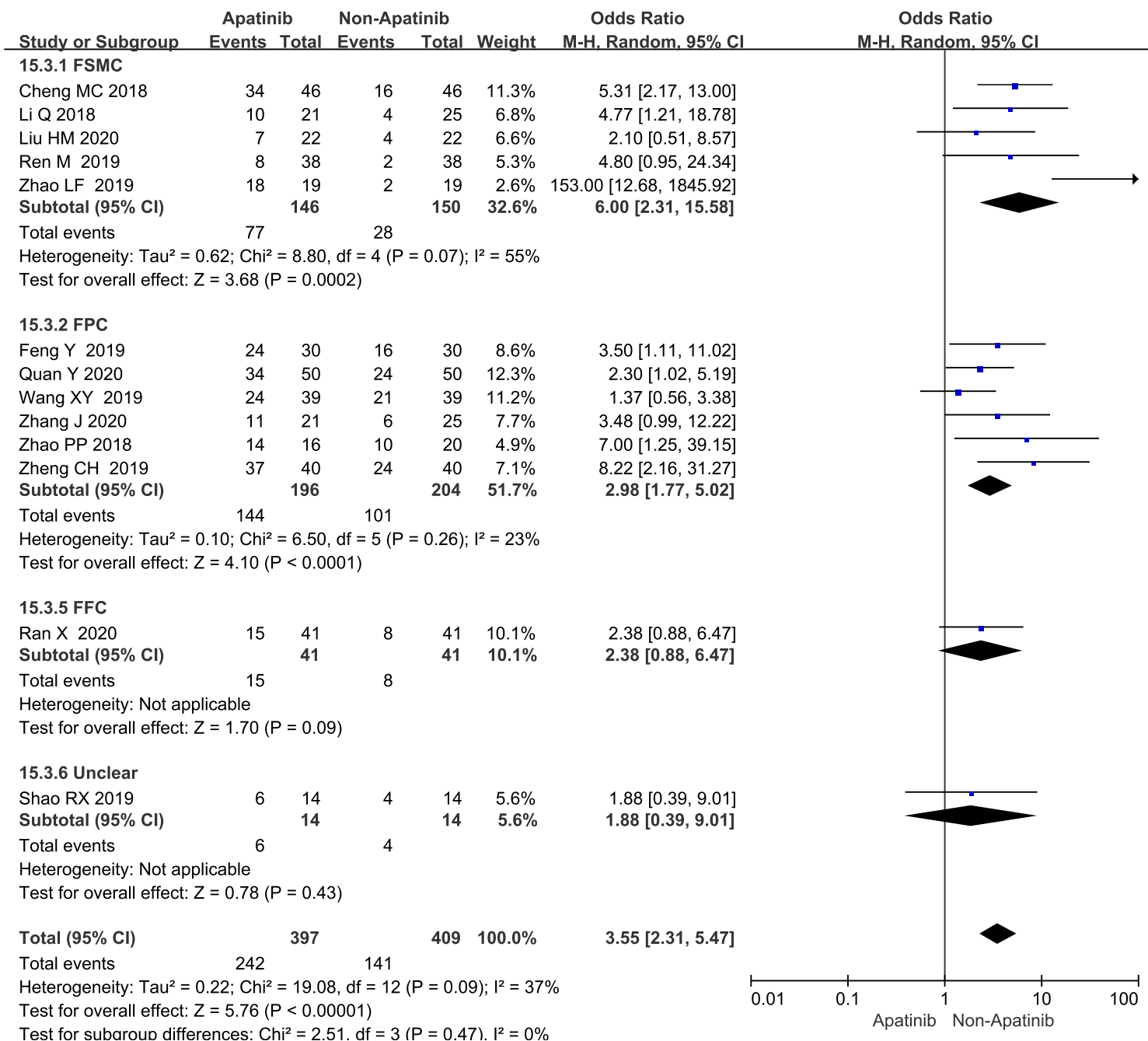

Supplement: Supplementary Materials — Supplementary Material 1. Figures S1–S21: forest plots of subgroup and metaregression analysis; Supplementary Material 2. Table S1 and Figures S22–32: results of publication bias analysis; and Supplementary Material 3. Figures S33–43: results of sensitivity analysis. [file 2292907.f1.zip › 2292907.f1/Figure S14 Subgroup analysis ORR according to treatment process.pdf]

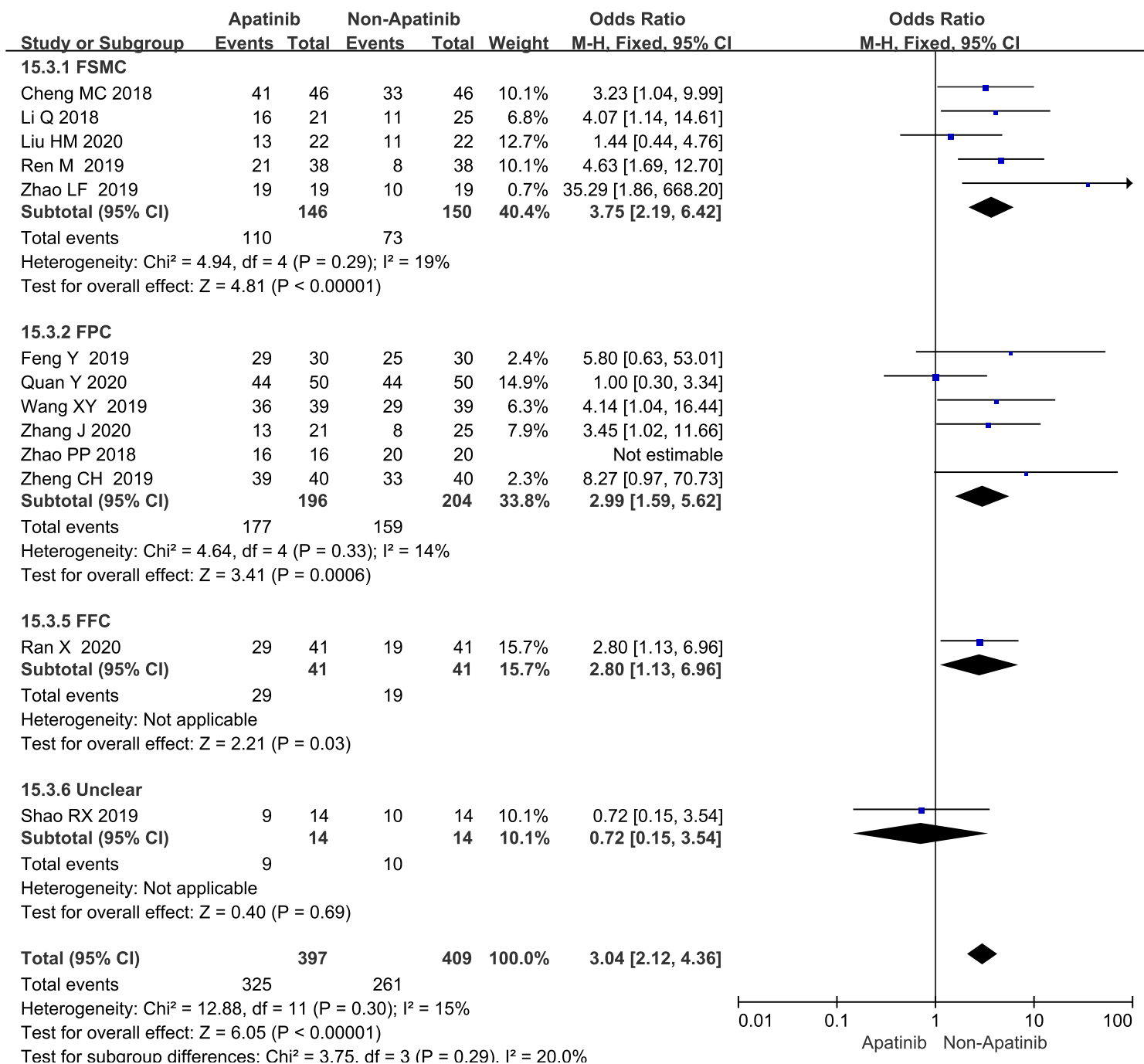

Supplement: Supplementary Materials — Supplementary Material 1. Figures S1–S21: forest plots of subgroup and metaregression analysis; Supplementary Material 2. Table S1 and Figures S22–32: results of publication bias analysis; and Supplementary Material 3. Figures S33–43: results of sensitivity analysis. [file 2292907.f1.zip › 2292907.f1/Figure S15 Subgroup analysis DCR according to treatment process.pdf]

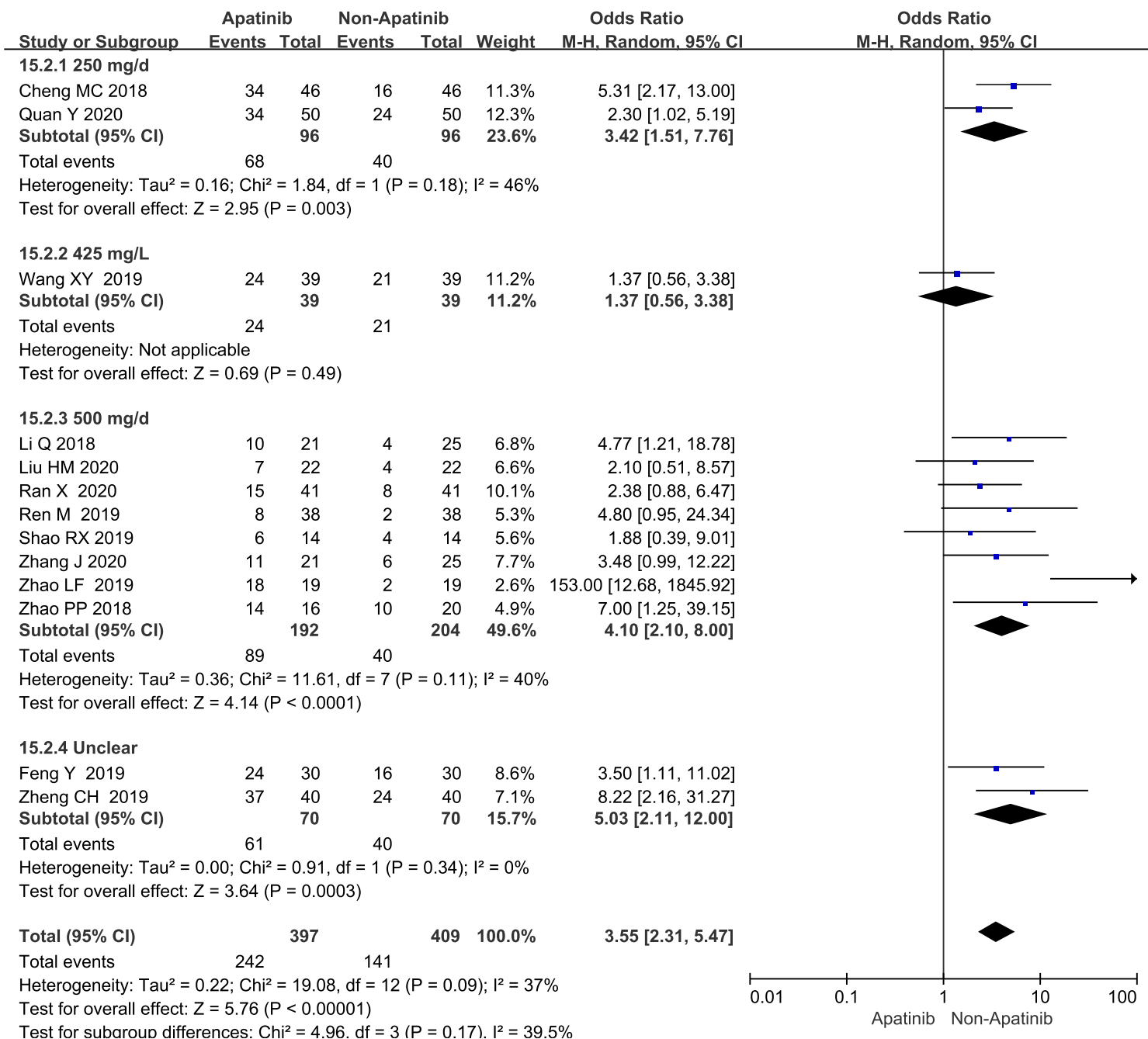

Supplement: Supplementary Materials — Supplementary Material 1. Figures S1–S21: forest plots of subgroup and metaregression analysis; Supplementary Material 2. Table S1 and Figures S22–32: results of publication bias analysis; and Supplementary Material 3. Figures S33–43: results of sensitivity analysis. [file 2292907.f1.zip › 2292907.f1/Figure S16 Subgroup analysis ORR according to dosage of Apatinib.pdf]

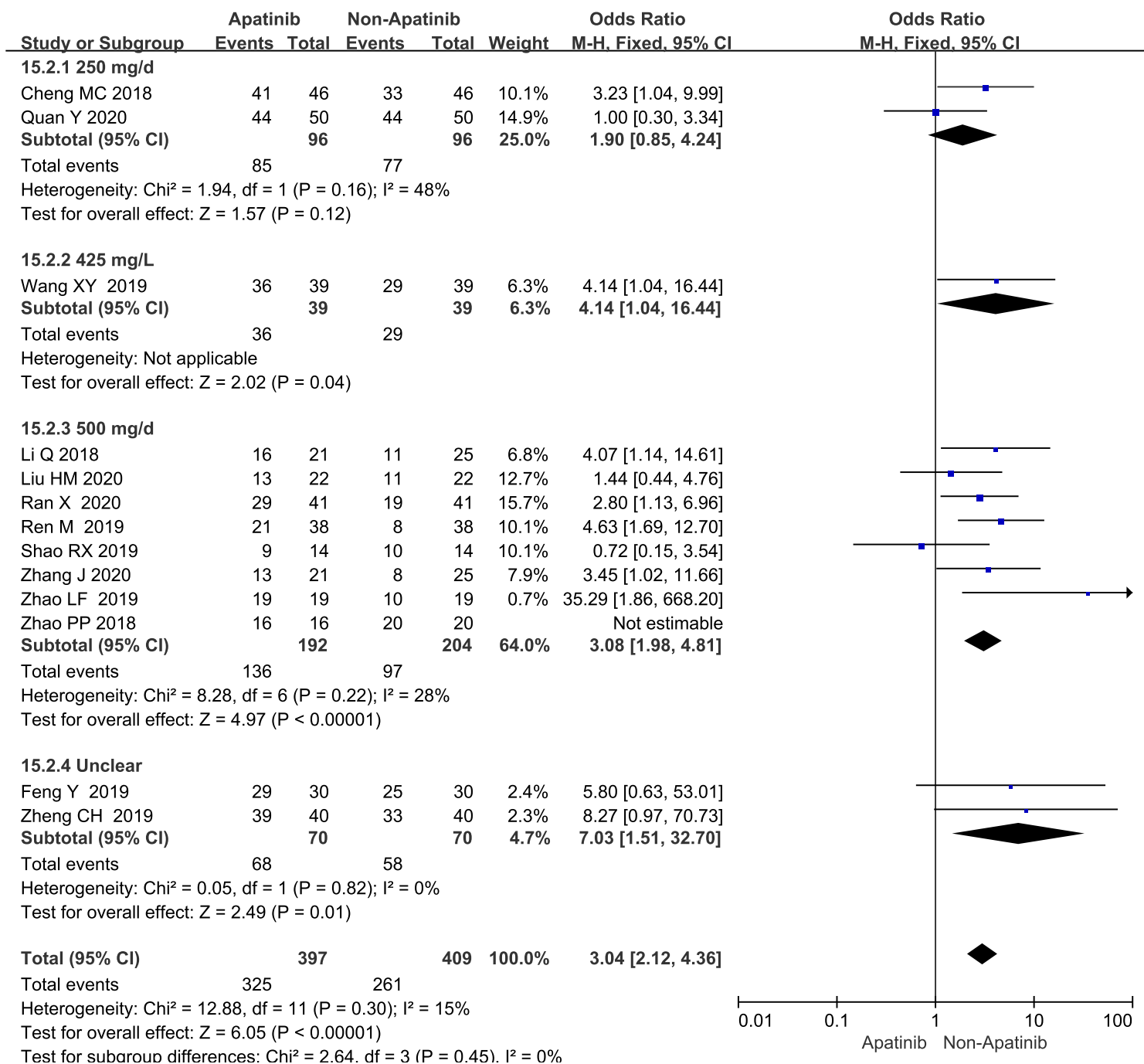

Supplement: Supplementary Materials — Supplementary Material 1. Figures S1–S21: forest plots of subgroup and metaregression analysis; Supplementary Material 2. Table S1 and Figures S22–32: results of publication bias analysis; and Supplementary Material 3. Figures S33–43: results of sensitivity analysis. [file 2292907.f1.zip › 2292907.f1/Figure S17 Subgroup analysis DCR according to dosage of Apatinib.pdf]

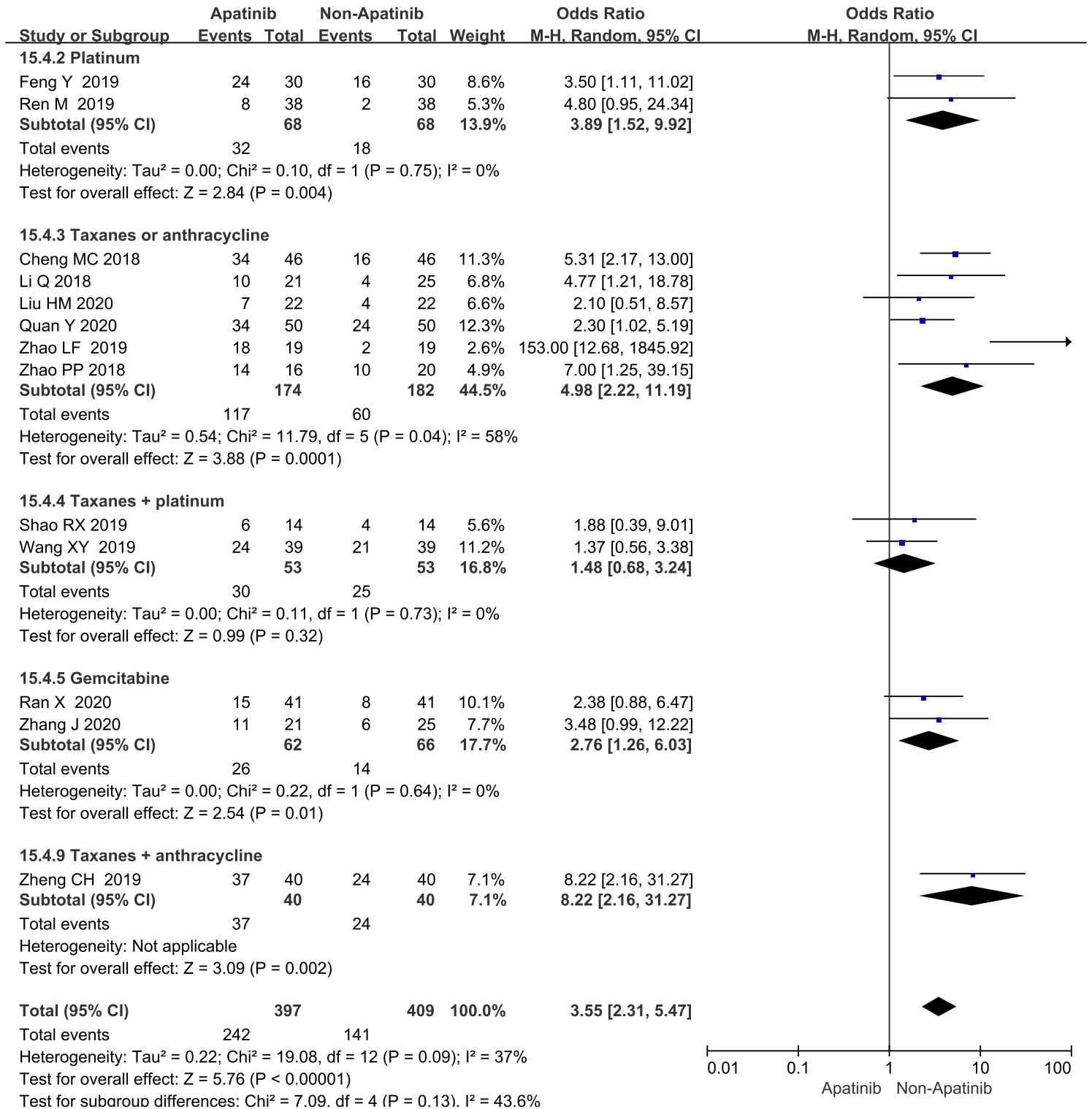

Supplement: Supplementary Materials — Supplementary Material 1. Figures S1–S21: forest plots of subgroup and metaregression analysis; Supplementary Material 2. Table S1 and Figures S22–32: results of publication bias analysis; and Supplementary Material 3. Figures S33–43: results of sensitivity analysis. [file 2292907.f1.zip › 2292907.f1/Figure S18 Subgroup analysis ORR according to chemotherapy regimen.pdf]

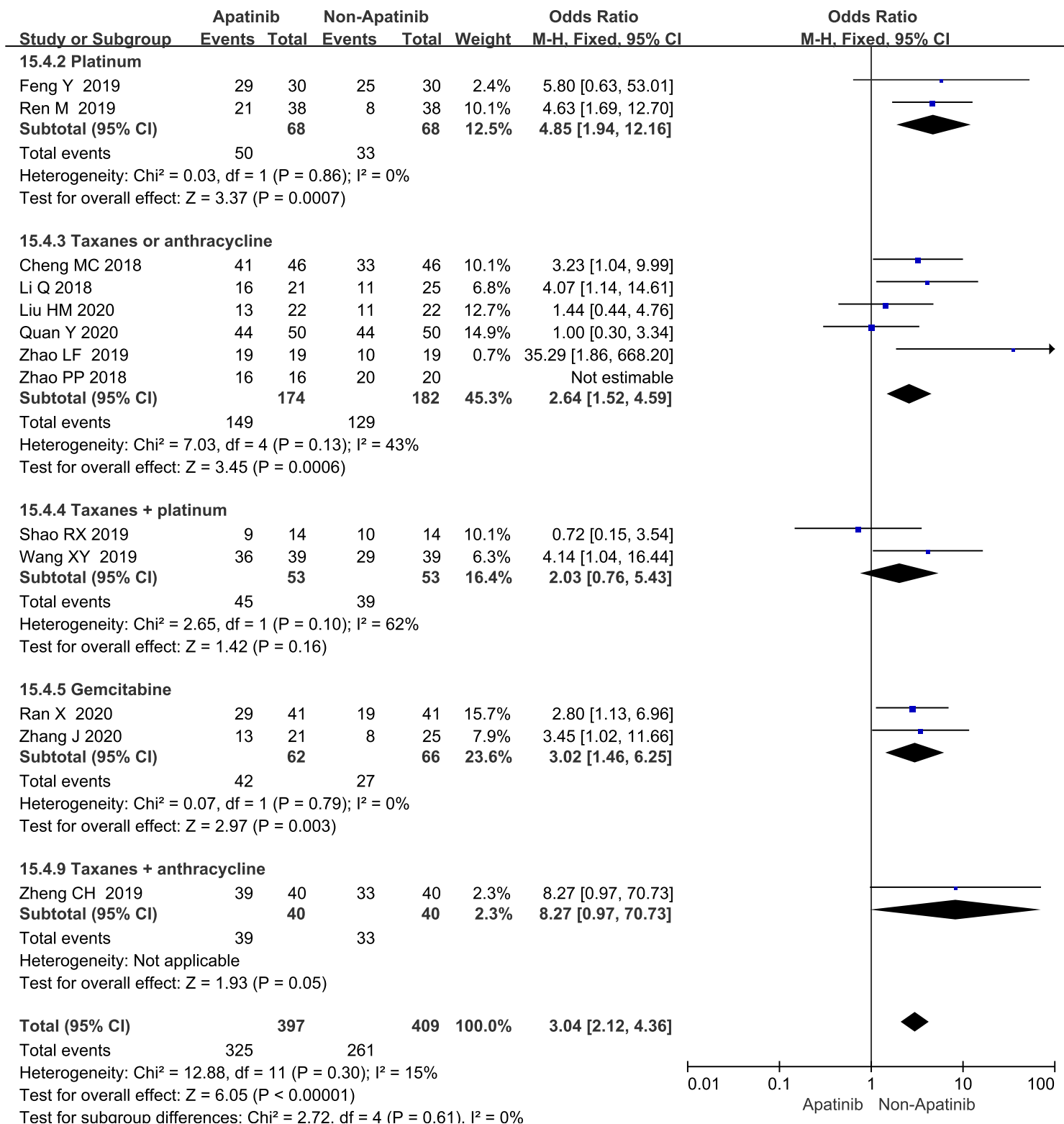

Supplement: Supplementary Materials — Supplementary Material 1. Figures S1–S21: forest plots of subgroup and metaregression analysis; Supplementary Material 2. Table S1 and Figures S22–32: results of publication bias analysis; and Supplementary Material 3. Figures S33–43: results of sensitivity analysis. [file 2292907.f1.zip › 2292907.f1/Figure S19 Subgroup analysis DCR according to chemotherapy regimen.pdf]

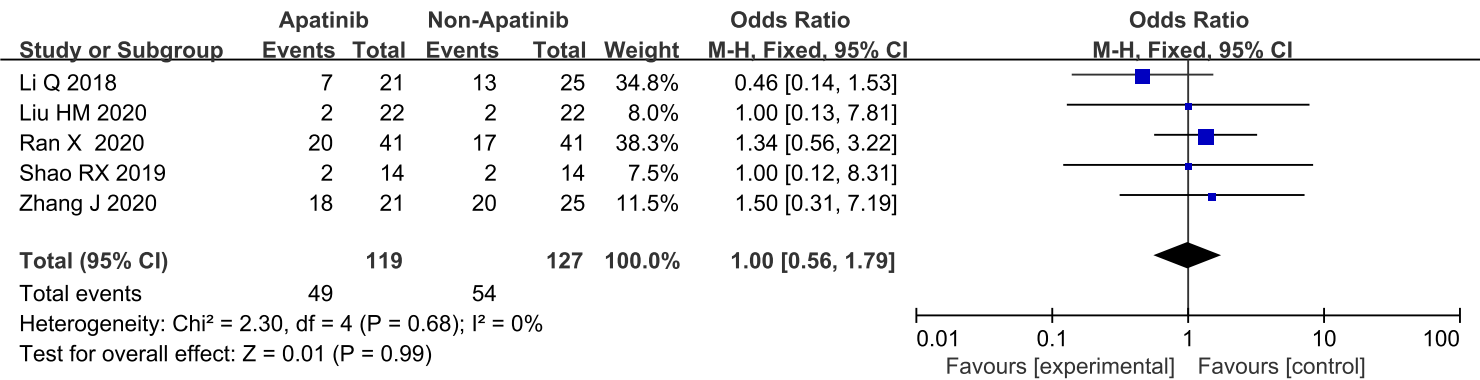

Supplement: Supplementary Materials — Supplementary Material 1. Figures S1–S21: forest plots of subgroup and metaregression analysis; Supplementary Material 2. Table S1 and Figures S22–32: results of publication bias analysis; and Supplementary Material 3. Figures S33–43: results of sensitivity analysis. [file 2292907.f1.zip › 2292907.f1/Figure S2 Meta-analysis results of leucopenia.pdf]

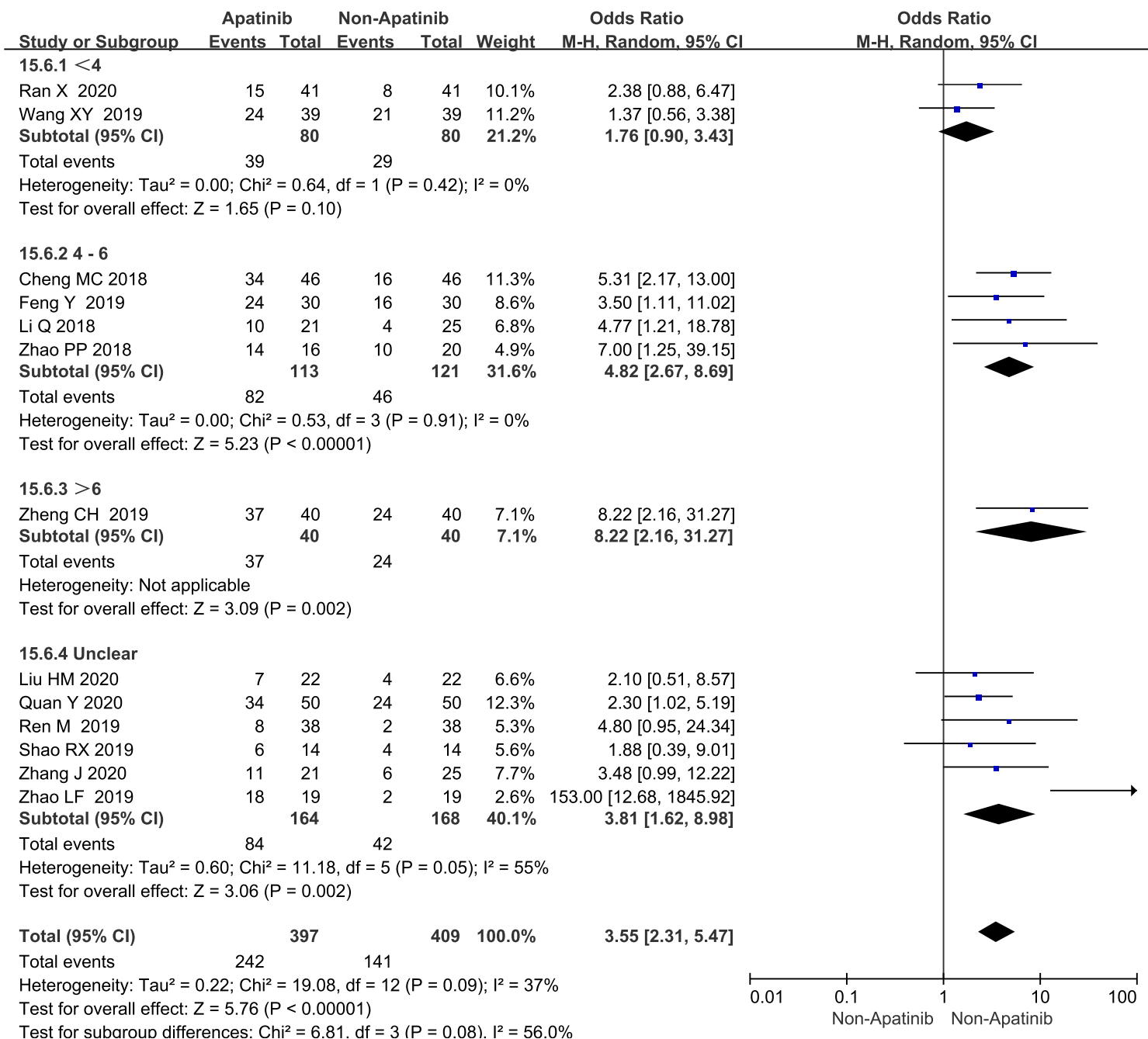

Supplement: Supplementary Materials — Supplementary Material 1. Figures S1–S21: forest plots of subgroup and metaregression analysis; Supplementary Material 2. Table S1 and Figures S22–32: results of publication bias analysis; and Supplementary Material 3. Figures S33–43: results of sensitivity analysis. [file 2292907.f1.zip › 2292907.f1/Figure S20 Subgroup analysis ORR according to chemotherapy cycle.pdf]

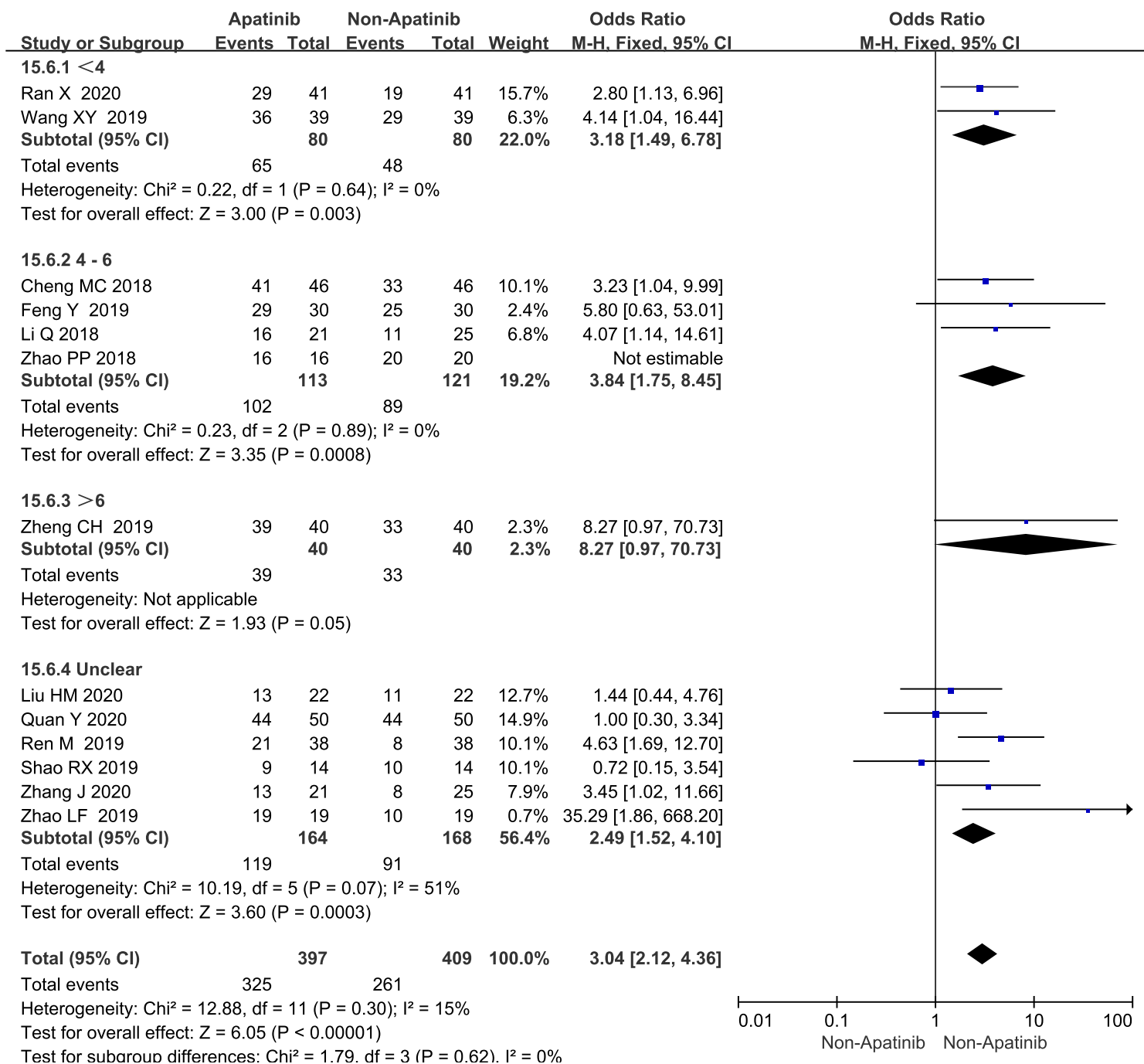

Supplement: Supplementary Materials — Supplementary Material 1. Figures S1–S21: forest plots of subgroup and metaregression analysis; Supplementary Material 2. Table S1 and Figures S22–32: results of publication bias analysis; and Supplementary Material 3. Figures S33–43: results of sensitivity analysis. [file 2292907.f1.zip › 2292907.f1/Figure S21 Subgroup analysis DCR according to chemotherapy cycle.pdf]

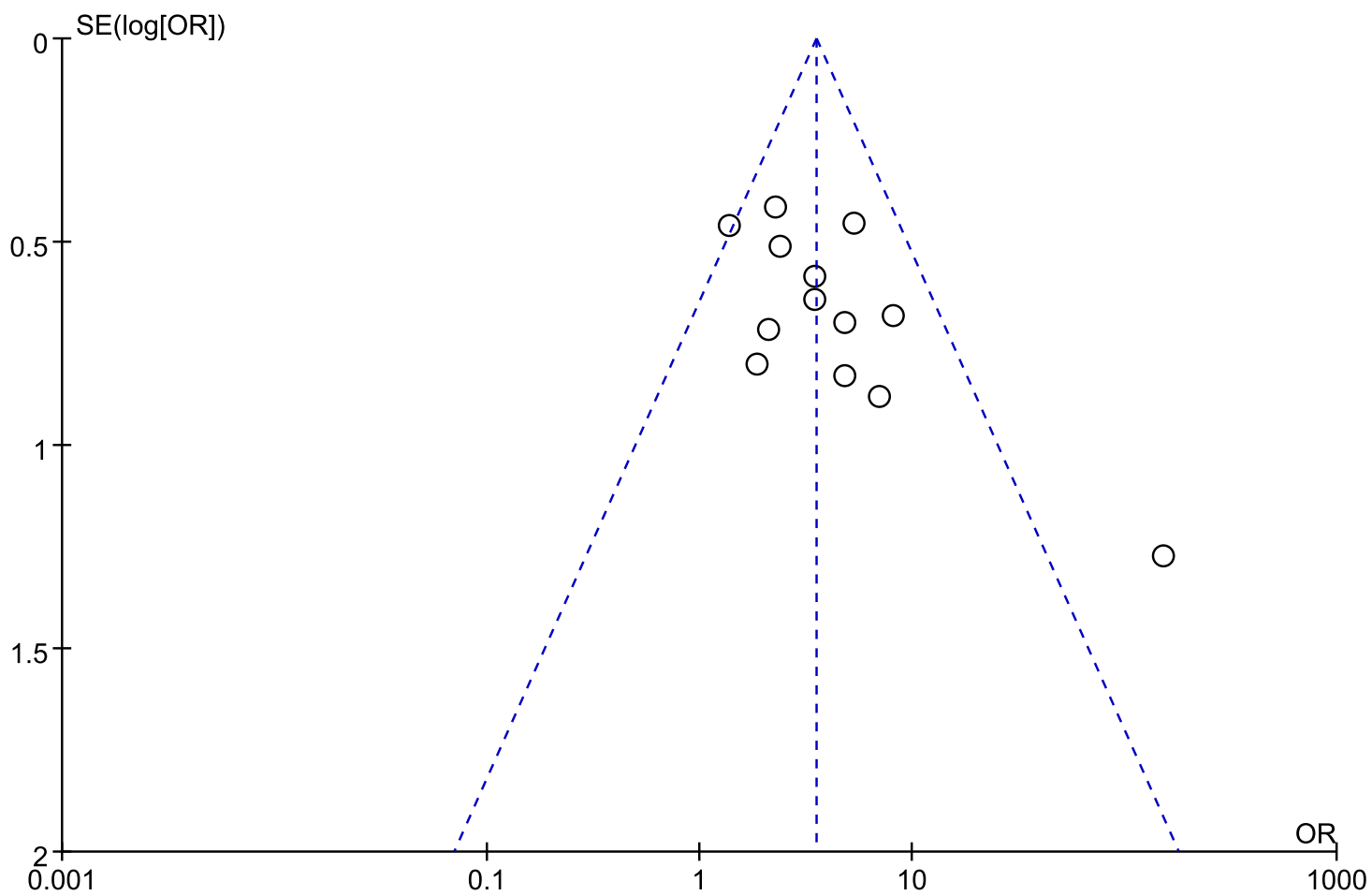

Supplement: Supplementary Materials — Supplementary Material 1. Figures S1–S21: forest plots of subgroup and metaregression analysis; Supplementary Material 2. Table S1 and Figures S22–32: results of publication bias analysis; and Supplementary Material 3. Figures S33–43: results of sensitivity analysis. [file 2292907.f1.zip › 2292907.f1/Figure S22 The funnel plot of objective response rate.pdf]

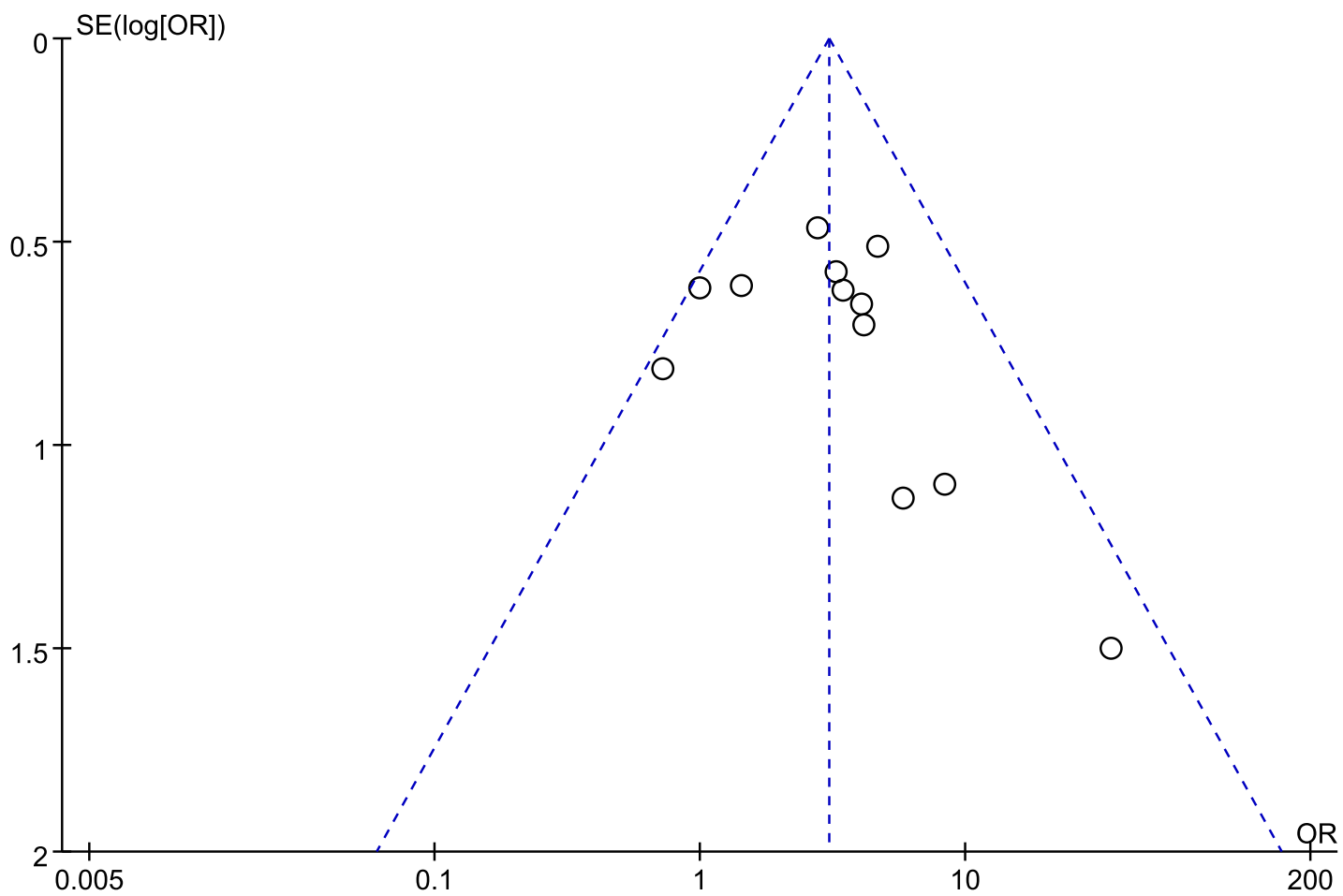

Supplement: Supplementary Materials — Supplementary Material 1. Figures S1–S21: forest plots of subgroup and metaregression analysis; Supplementary Material 2. Table S1 and Figures S22–32: results of publication bias analysis; and Supplementary Material 3. Figures S33–43: results of sensitivity analysis. [file 2292907.f1.zip › 2292907.f1/Figure S23 The funnel plot of disease control rate.pdf]

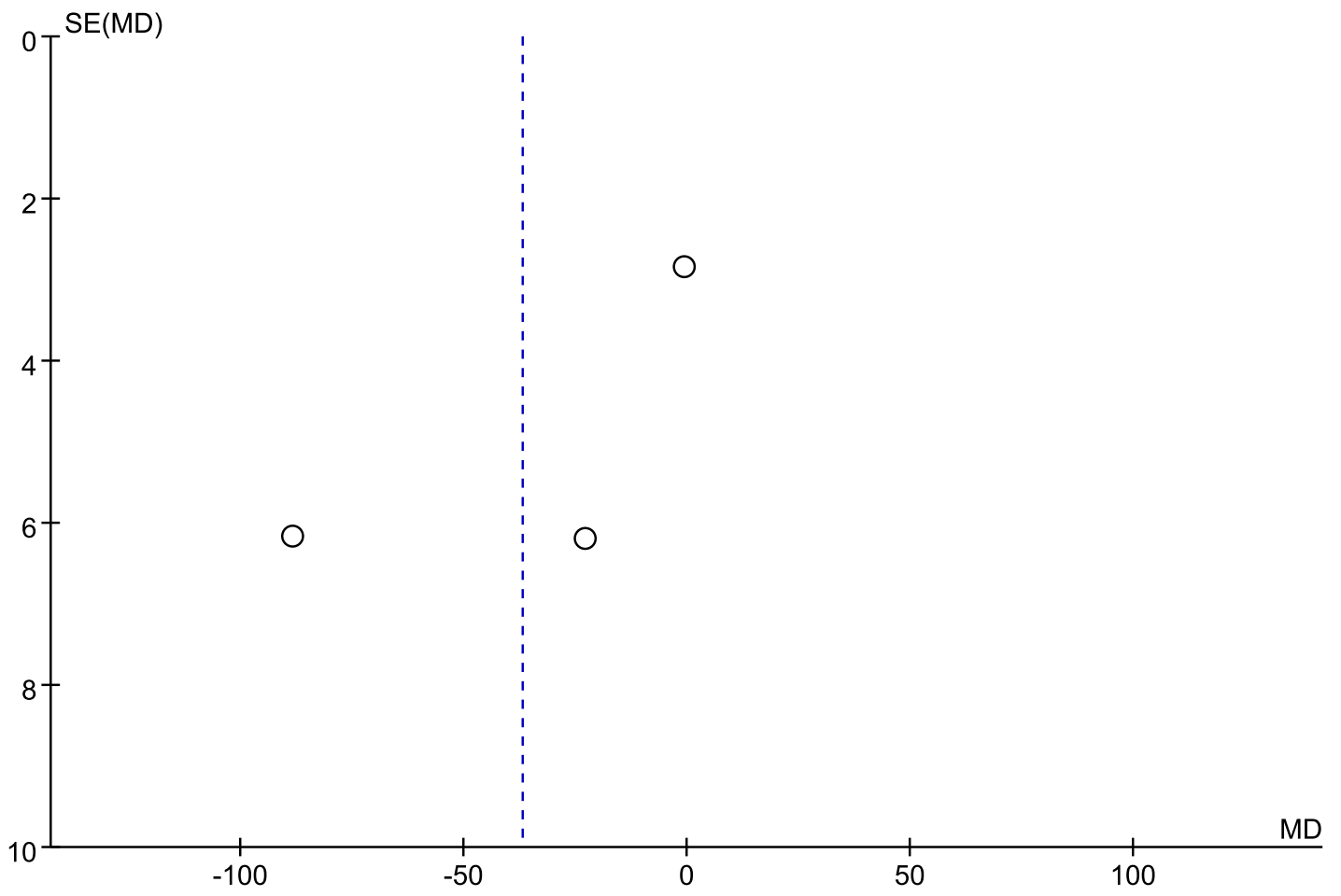

Supplement: Supplementary Materials — Supplementary Material 1. Figures S1–S21: forest plots of subgroup and metaregression analysis; Supplementary Material 2. Table S1 and Figures S22–32: results of publication bias analysis; and Supplementary Material 3. Figures S33–43: results of sensitivity analysis. [file 2292907.f1.zip › 2292907.f1/Figure S24 The funnel plot of the level of CA125.pdf]

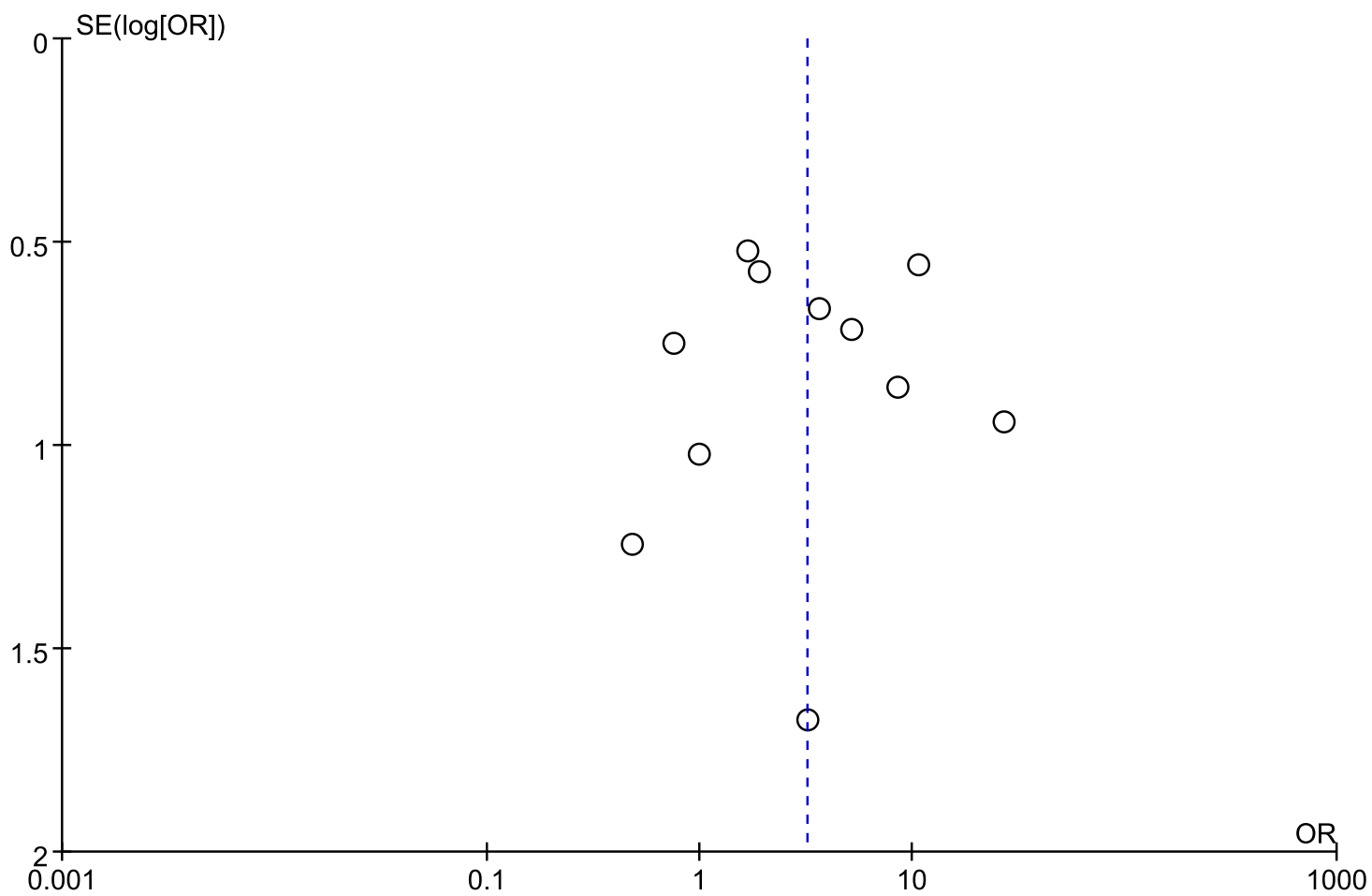

Supplement: Supplementary Materials — Supplementary Material 1. Figures S1–S21: forest plots of subgroup and metaregression analysis; Supplementary Material 2. Table S1 and Figures S22–32: results of publication bias analysis; and Supplementary Material 3. Figures S33–43: results of sensitivity analysis. [file 2292907.f1.zip › 2292907.f1/Figure S25 The funnel plot of myelosuppression.pdf]

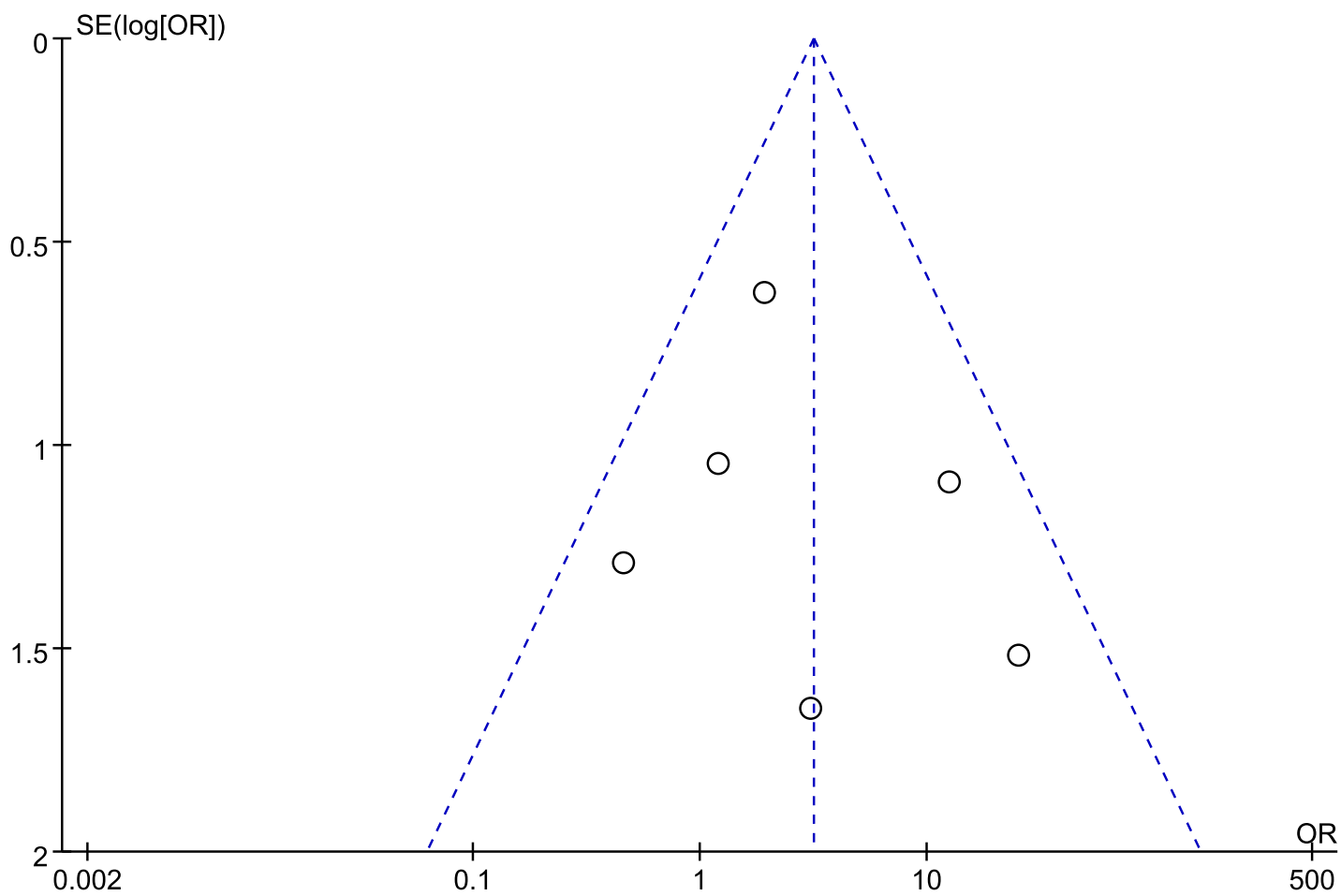

Supplement: Supplementary Materials — Supplementary Material 1. Figures S1–S21: forest plots of subgroup and metaregression analysis; Supplementary Material 2. Table S1 and Figures S22–32: results of publication bias analysis; and Supplementary Material 3. Figures S33–43: results of sensitivity analysis. [file 2292907.f1.zip › 2292907.f1/Figure S26 The funnel plot of hypertension.pdf]

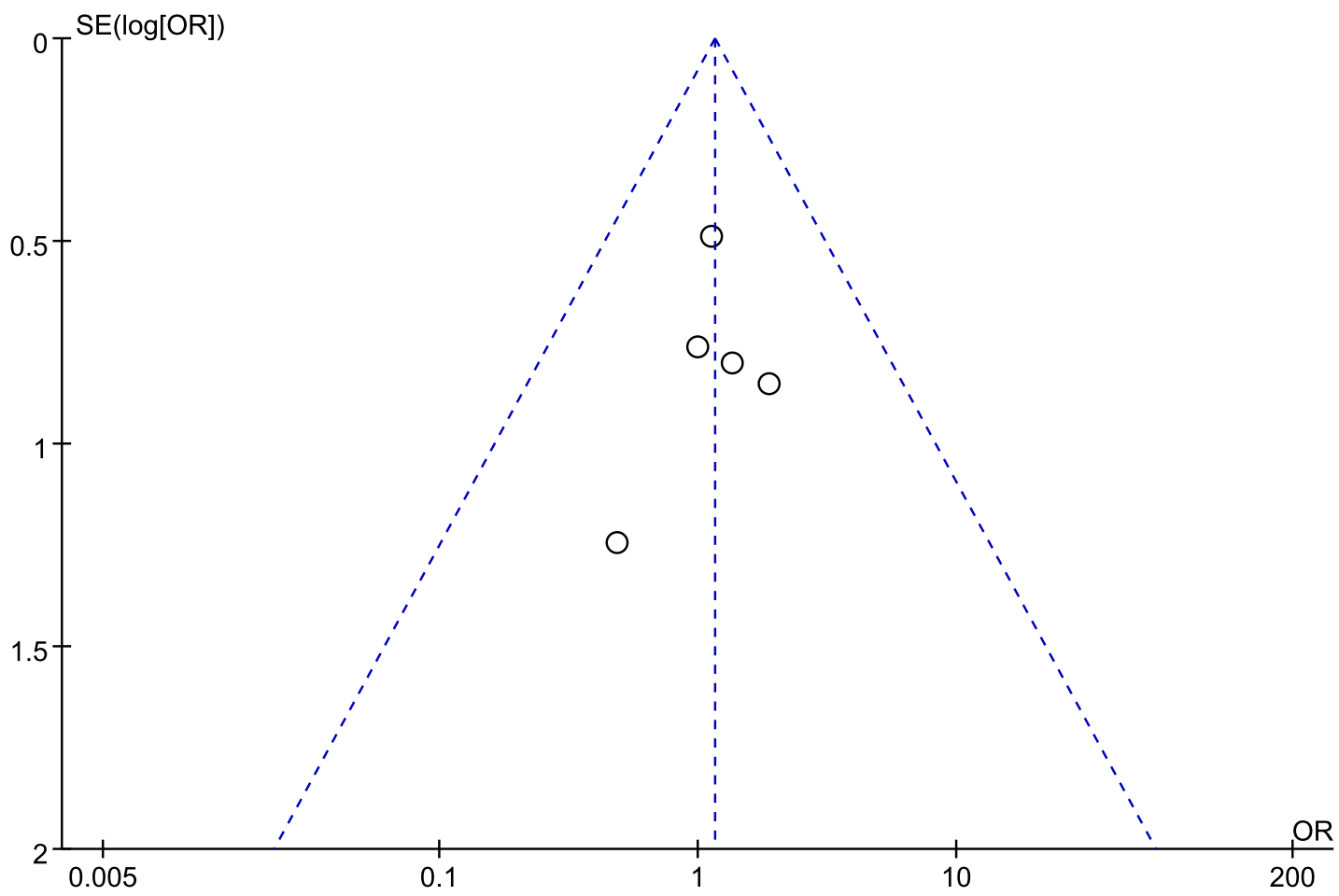

Supplement: Supplementary Materials — Supplementary Material 1. Figures S1–S21: forest plots of subgroup and metaregression analysis; Supplementary Material 2. Table S1 and Figures S22–32: results of publication bias analysis; and Supplementary Material 3. Figures S33–43: results of sensitivity analysis. [file 2292907.f1.zip › 2292907.f1/Figure S27 The funnel plot of proteinuria.pdf]

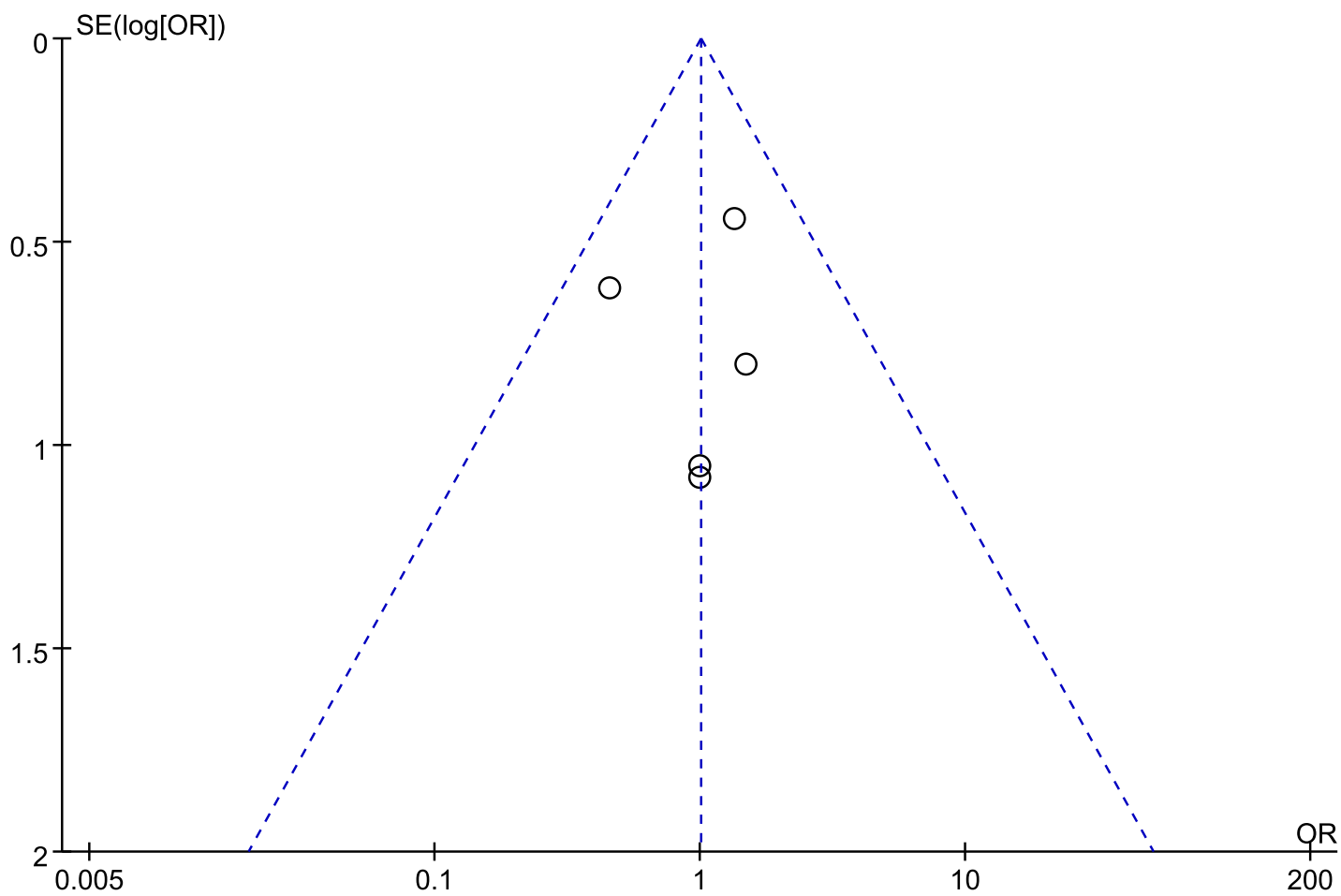

Supplement: Supplementary Materials — Supplementary Material 1. Figures S1–S21: forest plots of subgroup and metaregression analysis; Supplementary Material 2. Table S1 and Figures S22–32: results of publication bias analysis; and Supplementary Material 3. Figures S33–43: results of sensitivity analysis. [file 2292907.f1.zip › 2292907.f1/Figure S28 The funnel plot of leucopenia.pdf]

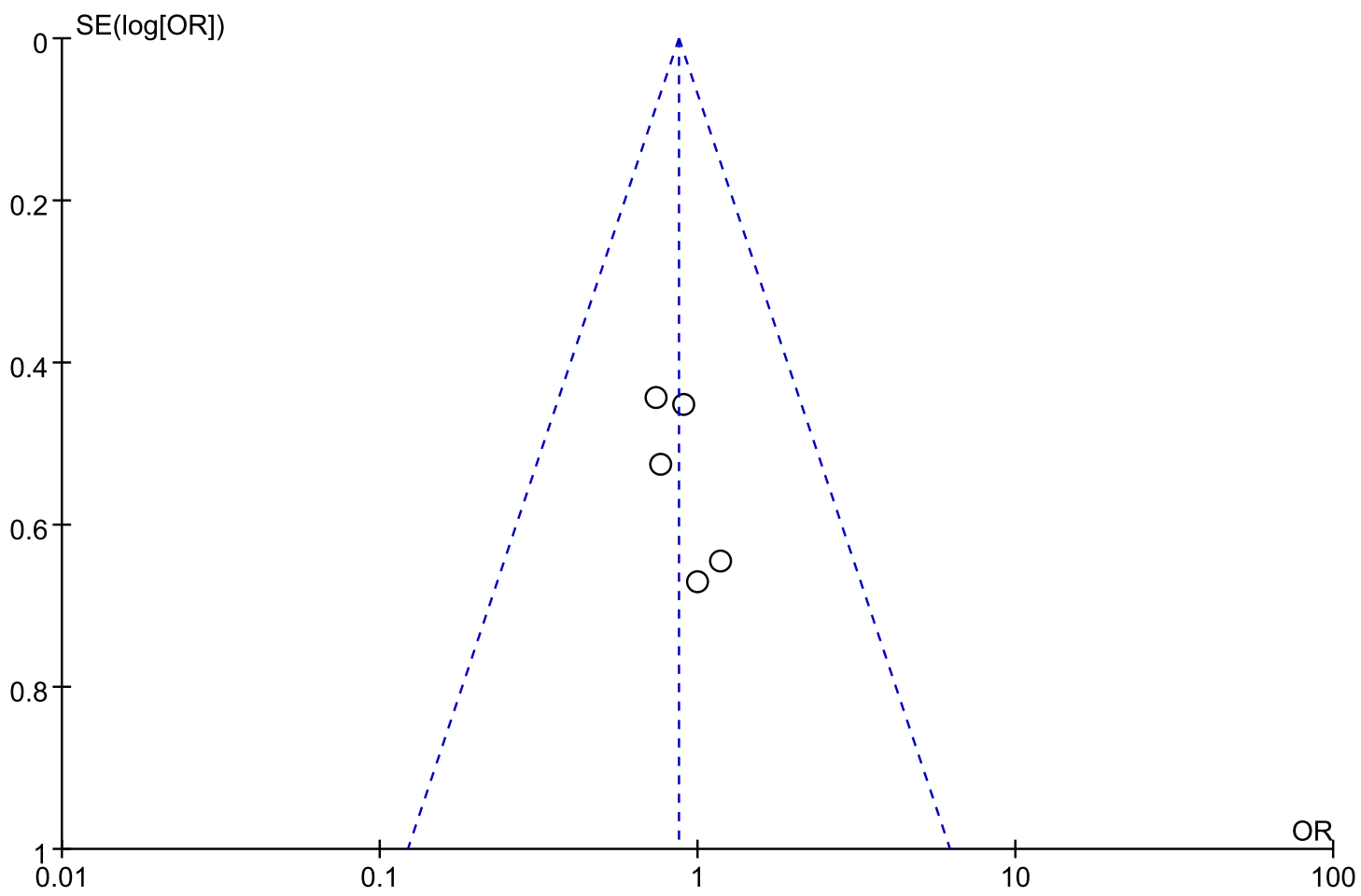

Supplement: Supplementary Materials — Supplementary Material 1. Figures S1–S21: forest plots of subgroup and metaregression analysis; Supplementary Material 2. Table S1 and Figures S22–32: results of publication bias analysis; and Supplementary Material 3. Figures S33–43: results of sensitivity analysis. [file 2292907.f1.zip › 2292907.f1/Figure S29 The funnel plot of gastrointestinal reaction.pdf]

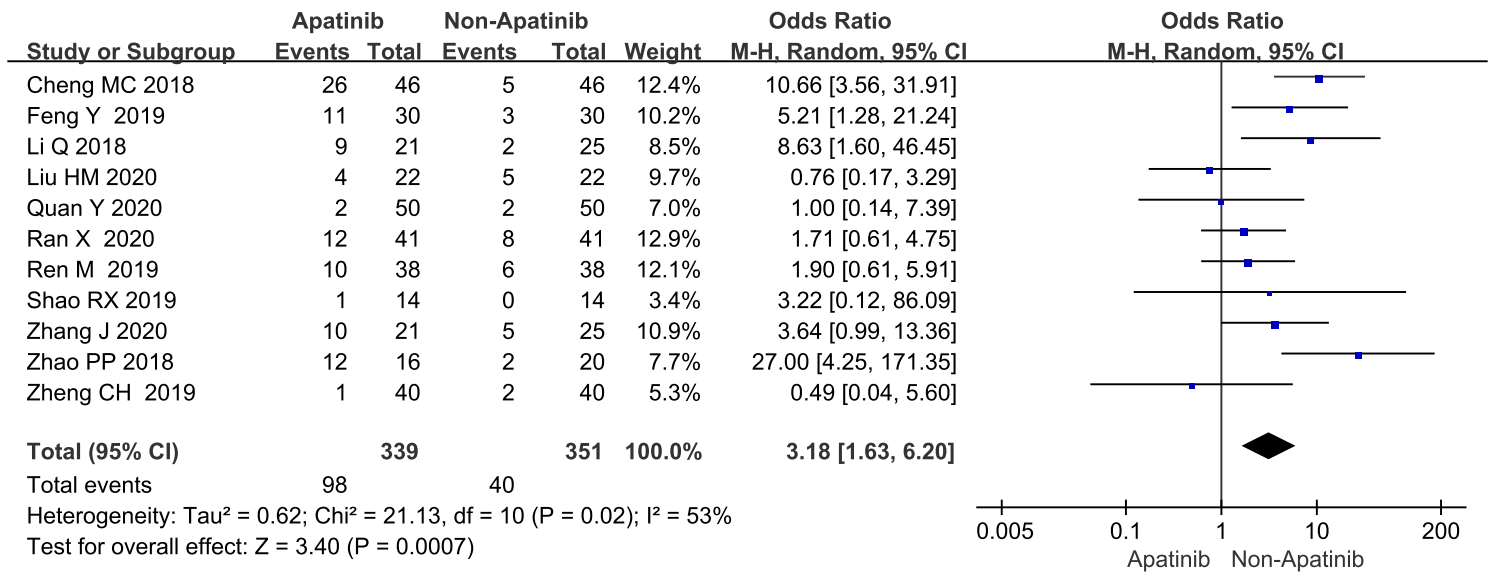

Supplement: Supplementary Materials — Supplementary Material 1. Figures S1–S21: forest plots of subgroup and metaregression analysis; Supplementary Material 2. Table S1 and Figures S22–32: results of publication bias analysis; and Supplementary Material 3. Figures S33–43: results of sensitivity analysis. [file 2292907.f1.zip › 2292907.f1/Figure S3 Meta-analysis results of hypertension.pdf]

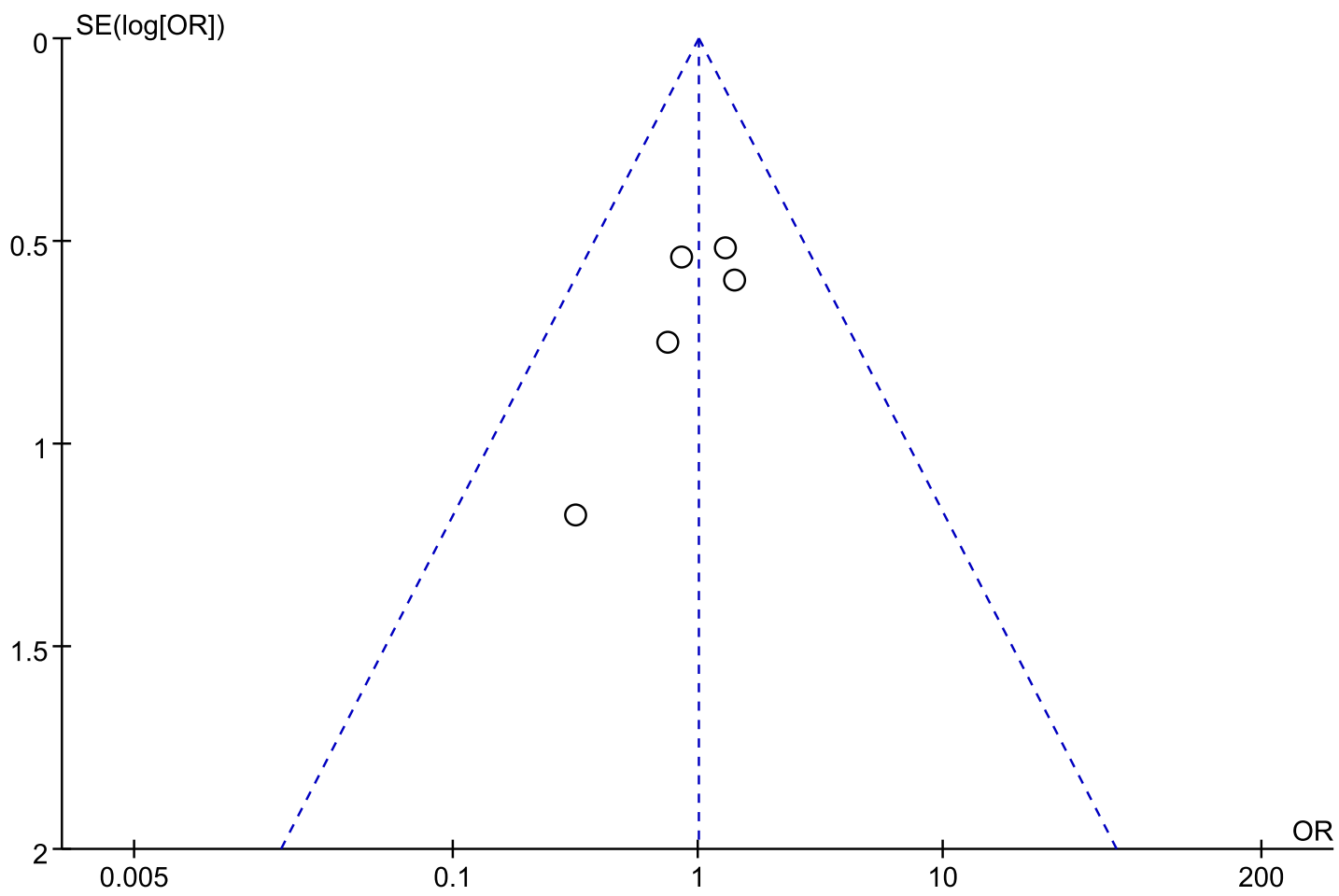

Supplement: Supplementary Materials — Supplementary Material 1. Figures S1–S21: forest plots of subgroup and metaregression analysis; Supplementary Material 2. Table S1 and Figures S22–32: results of publication bias analysis; and Supplementary Material 3. Figures S33–43: results of sensitivity analysis. [file 2292907.f1.zip › 2292907.f1/Figure S30 The funnel plot of nauseavomiting.pdf]

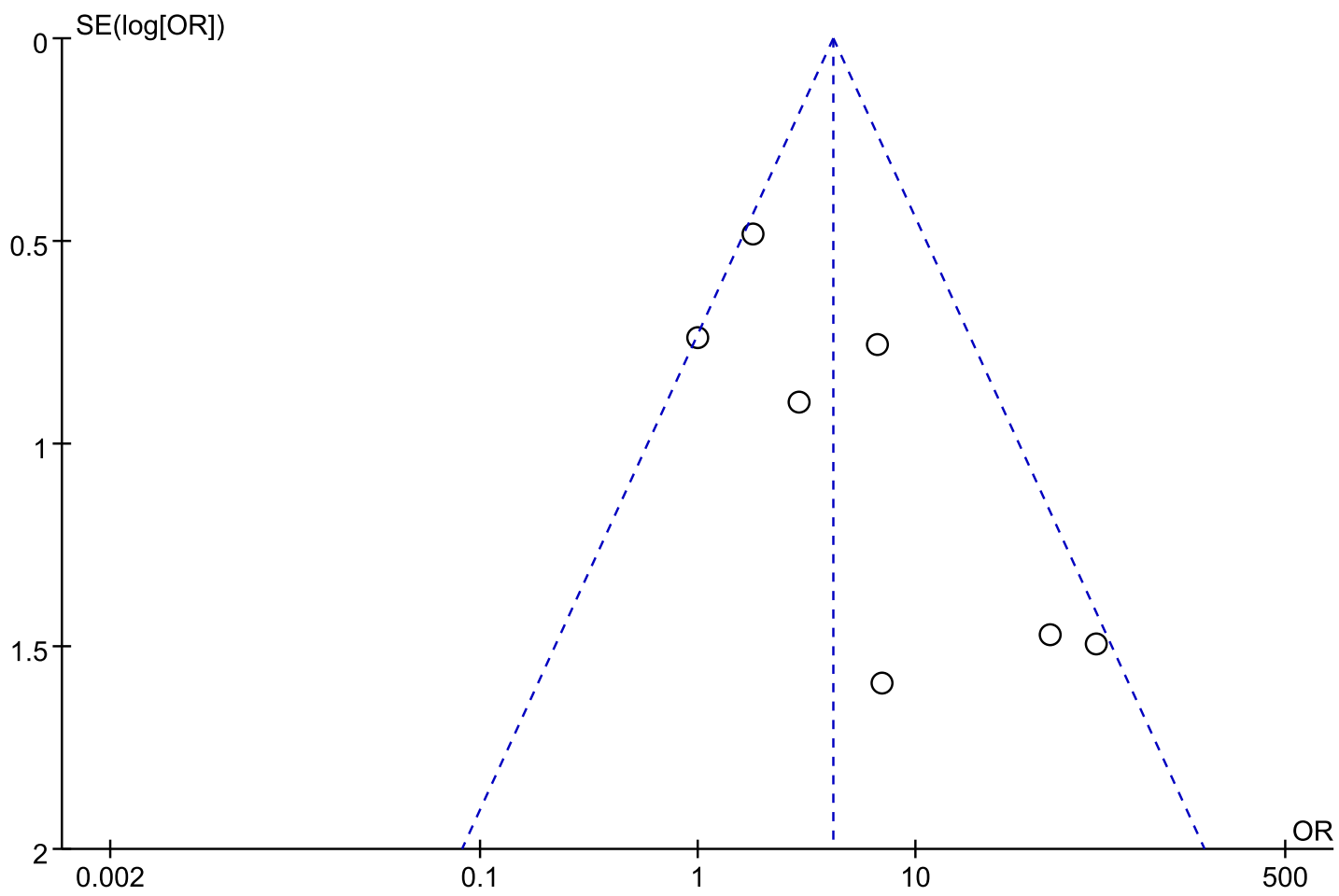

Supplement: Supplementary Materials — Supplementary Material 1. Figures S1–S21: forest plots of subgroup and metaregression analysis; Supplementary Material 2. Table S1 and Figures S22–32: results of publication bias analysis; and Supplementary Material 3. Figures S33–43: results of sensitivity analysis. [file 2292907.f1.zip › 2292907.f1/Figure S31 The funnel plot of hand-foot Syndrome.pdf]

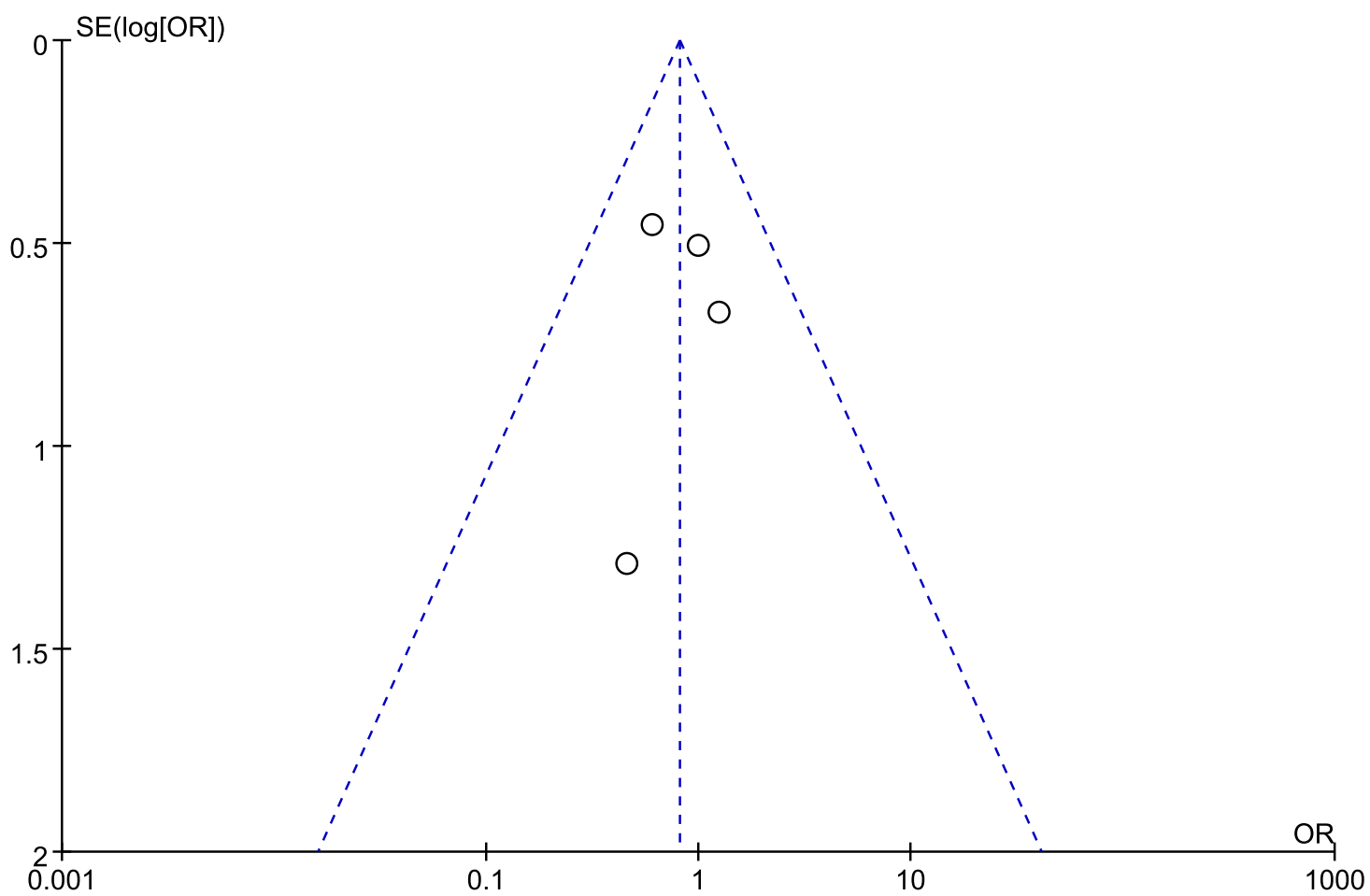

Supplement: Supplementary Materials — Supplementary Material 1. Figures S1–S21: forest plots of subgroup and metaregression analysis; Supplementary Material 2. Table S1 and Figures S22–32: results of publication bias analysis; and Supplementary Material 3. Figures S33–43: results of sensitivity analysis. [file 2292907.f1.zip › 2292907.f1/Figure S32 The funnel plot of liverrenal dysfunction..pdf]

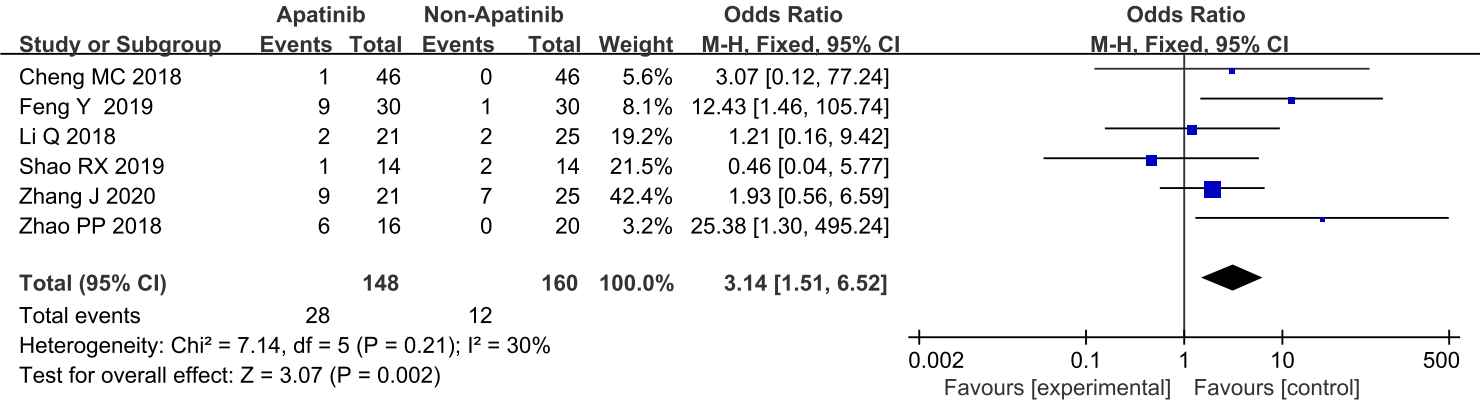

Supplement: Supplementary Materials — Supplementary Material 1. Figures S1–S21: forest plots of subgroup and metaregression analysis; Supplementary Material 2. Table S1 and Figures S22–32: results of publication bias analysis; and Supplementary Material 3. Figures S33–43: results of sensitivity analysis. [file 2292907.f1.zip › 2292907.f1/Figure S4 Meta-analysis results of proteinuria.pdf]

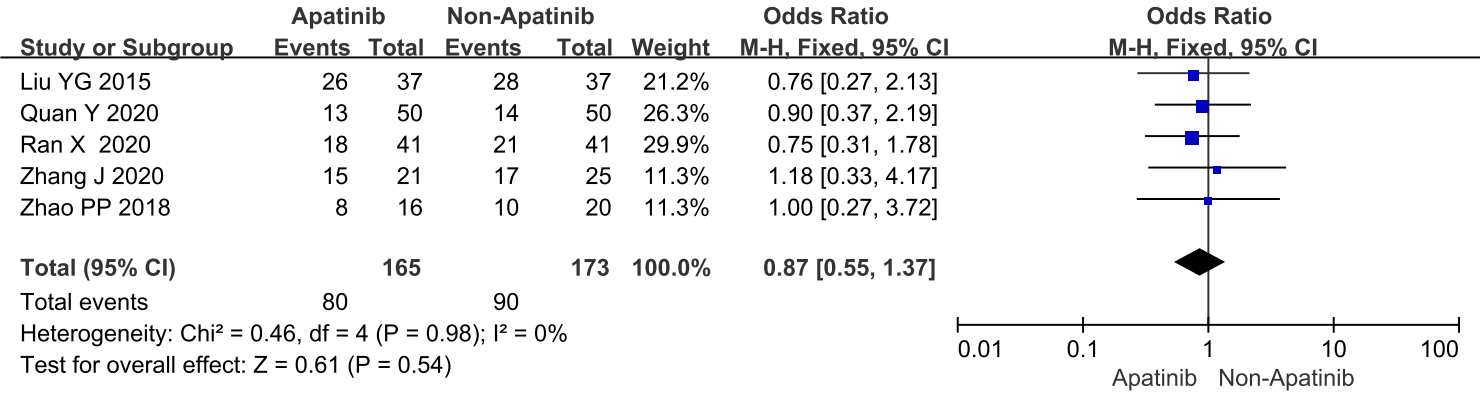

Supplement: Supplementary Materials — Supplementary Material 1. Figures S1–S21: forest plots of subgroup and metaregression analysis; Supplementary Material 2. Table S1 and Figures S22–32: results of publication bias analysis; and Supplementary Material 3. Figures S33–43: results of sensitivity analysis. [file 2292907.f1.zip › 2292907.f1/Figure S5 Meta-analysis results of gastrointestinal reaction.pdf]

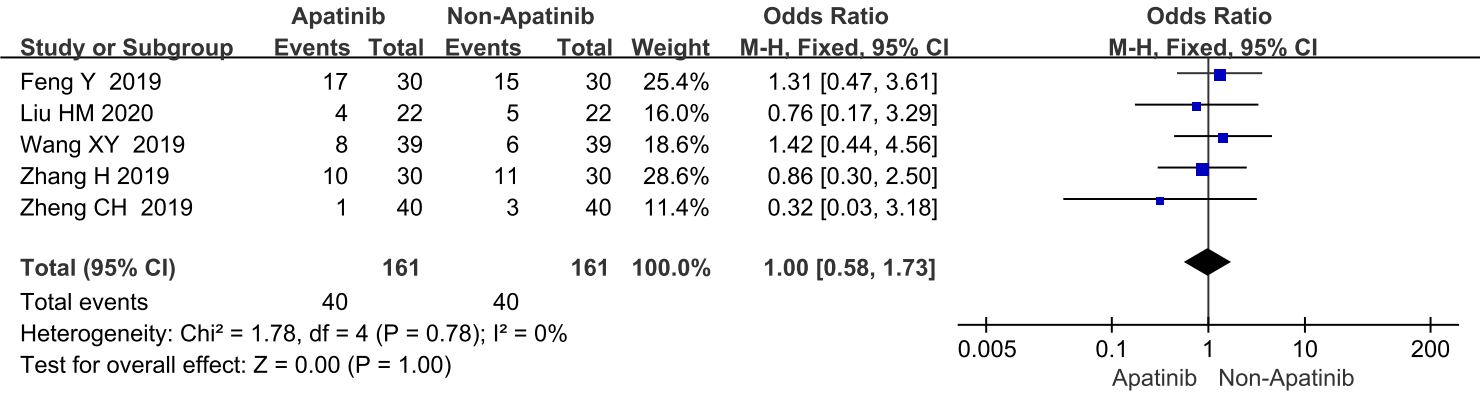

Supplement: Supplementary Materials — Supplementary Material 1. Figures S1–S21: forest plots of subgroup and metaregression analysis; Supplementary Material 2. Table S1 and Figures S22–32: results of publication bias analysis; and Supplementary Material 3. Figures S33–43: results of sensitivity analysis. [file 2292907.f1.zip › 2292907.f1/Figure S6 Meta-analysis results of nauseavomiting.pdf]

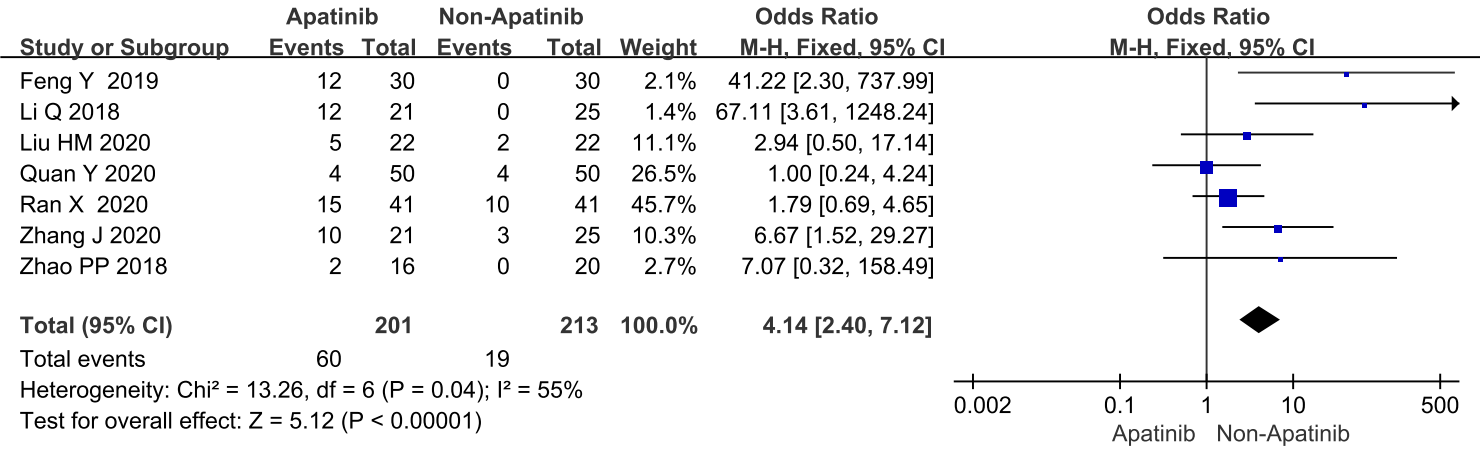

Supplement: Supplementary Materials — Supplementary Material 1. Figures S1–S21: forest plots of subgroup and metaregression analysis; Supplementary Material 2. Table S1 and Figures S22–32: results of publication bias analysis; and Supplementary Material 3. Figures S33–43: results of sensitivity analysis. [file 2292907.f1.zip › 2292907.f1/Figure S7 Meta-analysis results of hand-foot Syndrome.pdf]

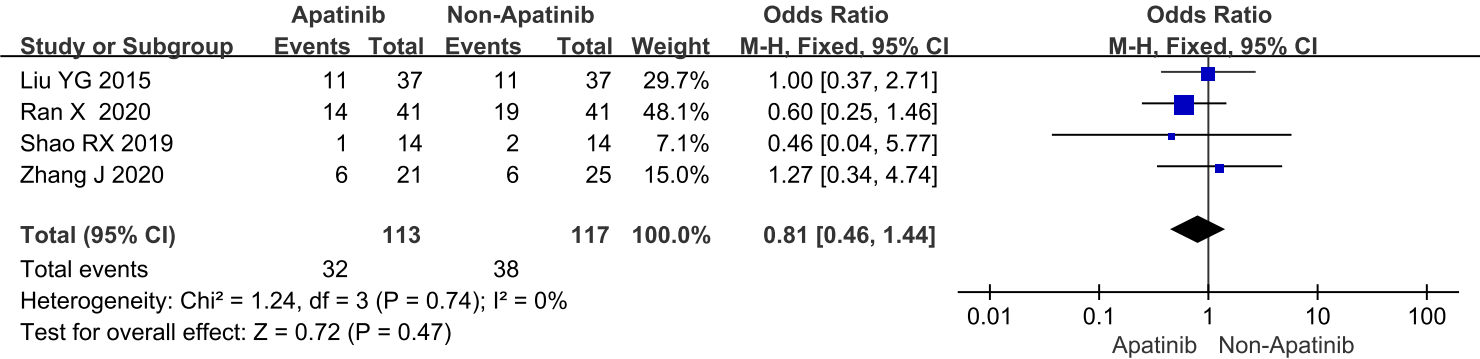

Supplement: Supplementary Materials — Supplementary Material 1. Figures S1–S21: forest plots of subgroup and metaregression analysis; Supplementary Material 2. Table S1 and Figures S22–32: results of publication bias analysis; and Supplementary Material 3. Figures S33–43: results of sensitivity analysis. [file 2292907.f1.zip › 2292907.f1/Figure S8 Meta-analysis results of liverrenal dysfunction.pdf]

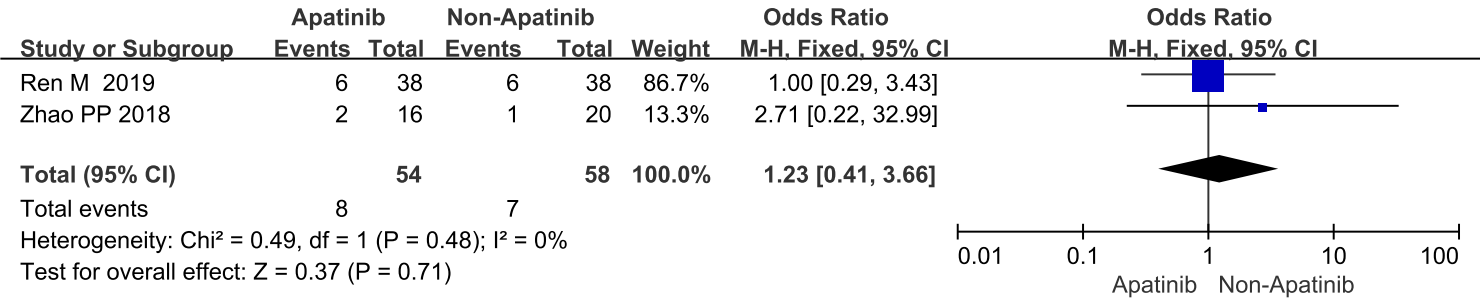

Supplement: Supplementary Materials — Supplementary Material 1. Figures S1–S21: forest plots of subgroup and metaregression analysis; Supplementary Material 2. Table S1 and Figures S22–32: results of publication bias analysis; and Supplementary Material 3. Figures S33–43: results of sensitivity analysis. [file 2292907.f1.zip › 2292907.f1/Figure S9 Meta-analysis results of fatigue reactions.pdf]
